# Extracellular vesicles cargo from head and neck cancer cell lines disrupt dendritic cells function and match plasma microRNAs

**DOI:** 10.1038/s41598-021-97753-y

**Published:** 2021-09-17

**Authors:** Elisangela de Paula Silva, Luciana Cavalheiro Marti, Flávia Maziero Andreghetto, Romário Oliveira de Sales, Martin Hoberman, Bárbara dos Santos Dias, Larissa Figueiredo Alves Diniz, Alessandro Marins dos Santos, Raquel Ajub Moyses, Otávio Alberto Curioni, Rossana Veronica Mendoza Lopez, Fabio Daumas Nunes, Eloiza Helena Tajara, Patricia Severino

**Affiliations:** 1 Centro de Pesquisa Experimental, Albert Einstein Research and Education Institute, Hospital Israelita Albert Einstein, Sao Paulo, Brazil; 2 Head and Neck Surgery Department, Hospital das Clínicas da Faculdade de Medicina, Universidade de São Paulo, Sao Paulo, Brazil; 3 Departamento de Cirurgia de Cabeça e Pescoço e Otorrinolaringologia, Hospital Heliópolis, Sao Paulo, Brazil; 4 Centro de Investigação Translacional em Oncologia, Instituto do Câncer do Estado de São Paulo, Sao Paulo, Brazil; 5 Department of Oral Pathology, School of Dentistry, Universidade de São Paulo, Sao Paulo, Brazil; 6 Department of Molecular Biology, Faculdade de Medicina de São José Do Rio Preto, Sao Paulo, Brazil

**Keywords:** Biological techniques, Cancer, Molecular biology, Biomarkers, Molecular medicine

## Abstract

Extracellular vesicles (EVs) are mediators of the immune system response. Encapsulated in EVs, microRNAs can be transferred between cancer and immune cells. To define the potential effects of EVs originated from squamous cell carcinoma cells on immune system response, we performed microRNA profiling of EVs released from two distinct cell lines and treated dendritic cells derived from circulating monocytes (mono-DCs) with these EVs. We confirmed the internalization of EVs by mono-DCs and the down-regulation of microRNA mRNA targets in treated mono-DCs. Differences in surface markers of dendritic cells cultivated in the presence of EVs indicated that their content disrupts the maturation process. Additionally, microRNAs known to interfere with dendritic cell function, and detected in EVs, matched microRNAs from squamous cell carcinoma patients’ plasma: miR-17-5p in oropharyngeal squamous cell carcinoma, miR-21 in oral squamous cell carcinoma, miR-16, miR-24, and miR-181a circulating in both oral and oropharyngeal squamous cell carcinoma, and miR-23b, which has not been previously described in plasma of head and neck squamous cell carcinoma, was found in plasma from patients with these cancer subtypes. This study contributes with insights on EVs in signaling between cancer and immune cells in squamous cell carcinoma of the head and neck.

## Introduction

As important components of the immune system, the main function of dendritic cells (DCs) is to initiate the adpative immune response through the processing of antigens and their presentation to T cells. Immature dendritic cells (iDCs) capture and process antigens and this physiological state is characterized by high levels of chemokine receptors (CCRs) and low levels of co-stimulatory molecules^1–3^. As iDCs migrate to lymphnodes, the maturation process is initiated and they acquire antigen presenting capabilities, a physiological state characterized by the secretion of cytokines and high expression of co-stimulatory molecules such as CD80, CD86 and MHC-II (HLA-DR)^4,5^. The differentiation is essential for DCs to become antigen presenting cells and activators of the Adaptive immune response^6,7^. Eventhough there are different classification systems for DCs and discussions concerning their origin, it is possible to reproduce their differentiation process in vitro using peripheral blood monocytes^8–10^.

The impairment of DC function has been widely studied in cancer. In fact, antigen recognition by T cells is one of the initial steps for the immune response against cancer, and a decrease in the frequency of mature DCs (mDCs) in cancer tissue has already been described, including for head and neck squamous cell carcinomas (HNSCC)^11–13^.

HNSCC is a heterogeneous disease that comprises a variety of tumors originating from the epithelial lining the oral and nasal cavities, larynx and pharynx^14^. In the United States new cases may reach 54,010 in 2021, with 10,850 deaths associated with cancers from the oral cavity and pharynx alone^15^. Heavy use of tobacco and alcohol as well as human papilloma virus infection (HPV) are among the most important risk factors for this disease^16,17^. Treatment is currently based on cancer staging at the time of diagnosis and includes surgery, chemotherapy and radiotherapy, with chemo and radiotherapy used either as single therapies or in association^18,19^. It has been described that immunosuppression significantly contributes to the progression of this cancer type^20^.

Among the molecular alterations described for HNSCC, microRNAs (miRNAs) have been extensively studied. MiRNAs are small non-coding RNAs implicated in gene expression regulation^21,22^. They are expressed in every human tissue but may also be found in body fluids, including serum and plasma, often associated with extracellular vesicles (EVs)^23–25^. EVs include exosomes and microvesicles, which originate from the cell endosomal system or that are shed from the plasma membrane, respectively. In cancer, EVs and their content, including miRNAs, have been studied in the context of cancer progression and immune system modulation^26–28^. For instance, EVs content transferred to DCs has been shown to alter MHC class II expression by immune cells with ensuing immune suppression^29^. The comprehension of the complexity of cell signaling processes and immune system modulation mediated by EVs demands proper cell models and may be dependent on the studied cancer type^30^.

In this study, monocyte-derived dendritic cells (mono-DCs) were treated with EVs derived from oropharyngeal squamous cell carcinoma (OPHSCC) and oral squamous cell carcinoma (OSCC) cell lines. We investigated if EVs derived from cell lines could serve as useful models for studying cell signaling from HNSCC cells to immune cells by comparing EVs content with plasma miRNAs from HNSCC patients. Results indicate impairment of mono-DC maturation and migration following EV internalization, and miRNAs carried by EVs were shown to match plasma miRNAs from HNSCC patients, a subset of which target biological processes associated with immune response.

## Results

### EVs collected from SCC cell lines culture medium are diverse in size and include miRNAs detected in plasma from HNSCC patients

**Figure 1:**
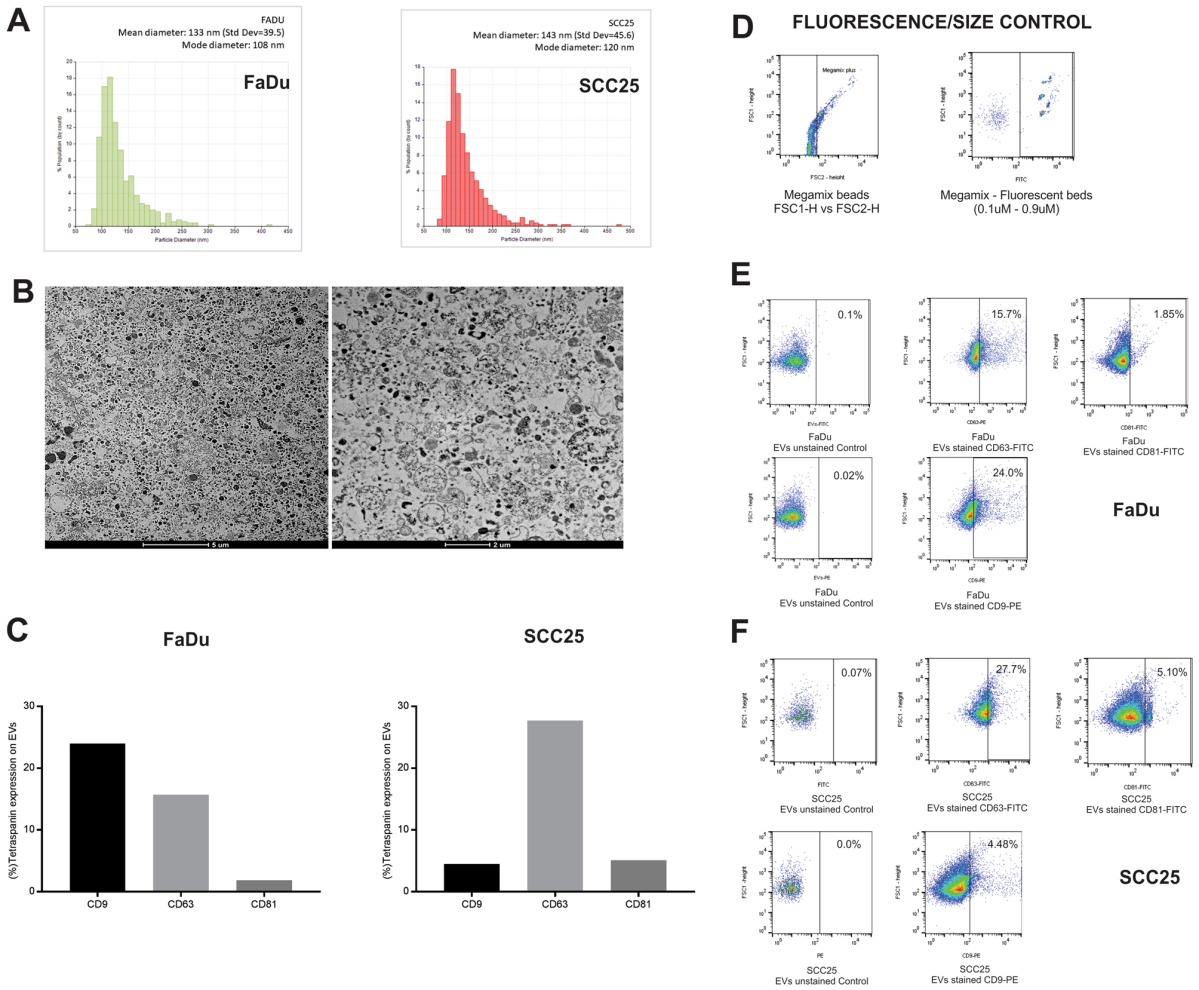
EVs characterization by size, imaging and tetraspanin expression. (**A**) Size distribution of EVs in SCC25 and FaDu culture medium. EV sizes were plotted over calibration particles diameters; (**B**) Representative images of transmission electron microscopy of EVs isolated from cell culture medium at × 2100 and × 3500 magnification, the selected images were obtained from FaDu culture medium but identical results were obtained for SCC25; (**C**) First graph displays the percentage of tetraspanin expression (CD9, CD63 and CD81) by EVs derived from FaDu cell lines and second graph displays the percentage of tetraspanin expression (CD9, CD63 and CD81) by EVs derived from SSC25 cell lines; (**D**) Flow cytometry control of fluorescence and size determination using Megamix-Plus FSC fluorescent particles with different sizes (0.1–0.9 µM); (**E**) Representative dot plot graphs displaying fow cytometry analysis of tetraspanin (CD9, CD63 and CD81) expression by EVs derived from FaDu cell line; (**F**) Representative dot plot graphs displaying fow cytometry analysis of tetraspanin expression by EVs derived from SCC25 cell line.

The size distribution of EVs isolated from the culture media of SCC25 and FaDu cell lines was characterized by TRPS (Fig. 1A), and EVs were visualized by transmission electron microscopy (Fig. 1B); a variety of sizes, illustrating the heterogeneity of vesicles, was observed, corroborating previous reports^31–34^. In addition, flow cytometry confirmed that a subset of the isolated EVs expressed the tetraspanins CD9, CD63 and CD81 (Fig. 1C–F), the most frequently identified proteins in exosomes.

**Figure 2:**
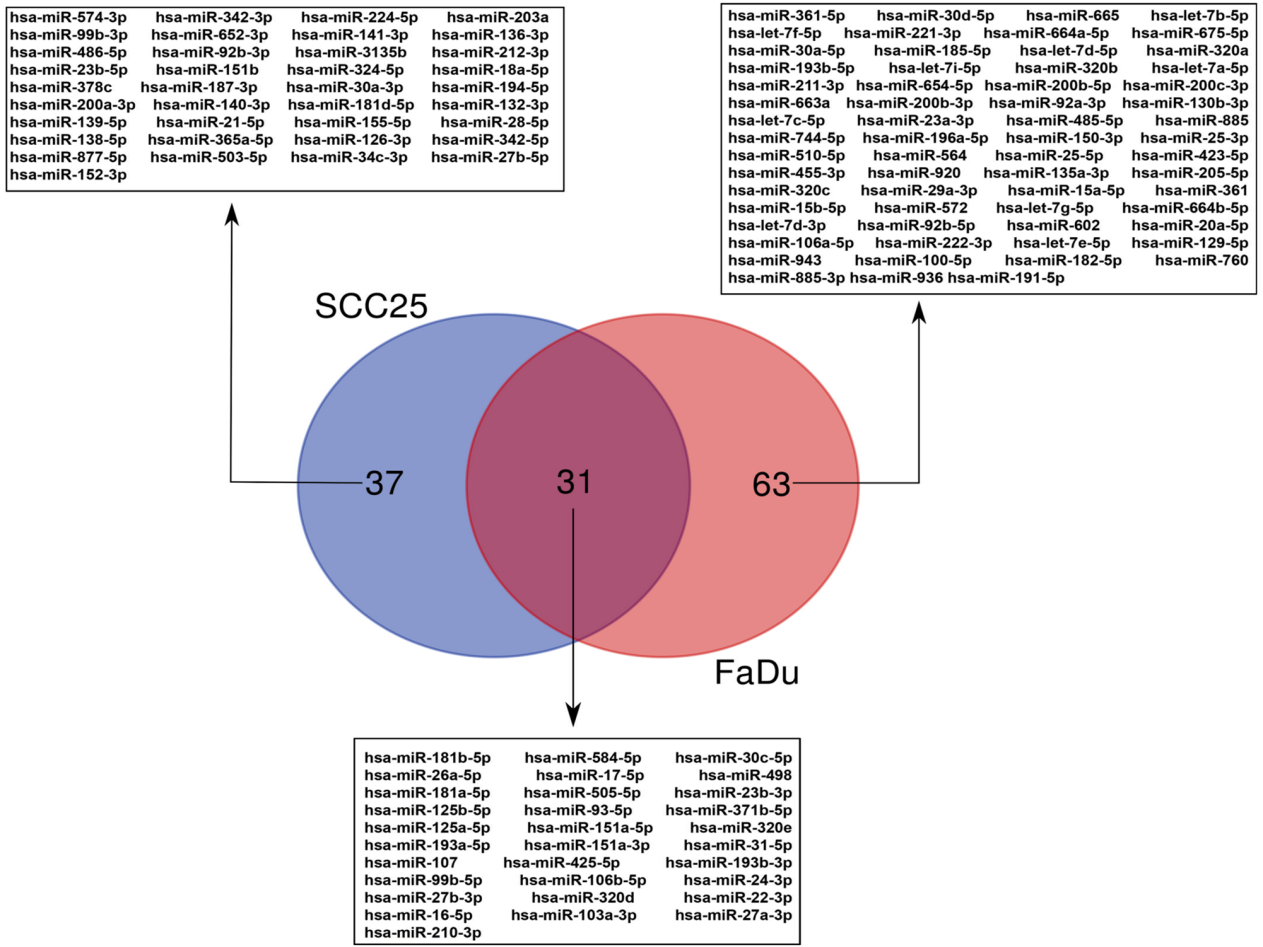
Venn diagram showing miRNAs detected in EVs isolated from FaDu and SCC25 cell culture medium using microarray assays.

MiRNA profiling of EVs was carried out using microarray assays. We were able to detect 96 miRNAs in EVs from FaDu and 68 miRNAs in EVs from SCC25, with 31 miRNAs common to both cell lines (Fig. 2 and Supplementary Table S1). According to current literature, of the miRNAs detected within EVs, 51 from FaDu and 24 miRNAs from SCC25 had been previously detected in plasma of OPHSCC and OSCC patients, respectively, the cancer subsites from which these cell lines derive (Supplementary Table S2).

### EVs are incorporated by mono-DCs

**Figure 3:**
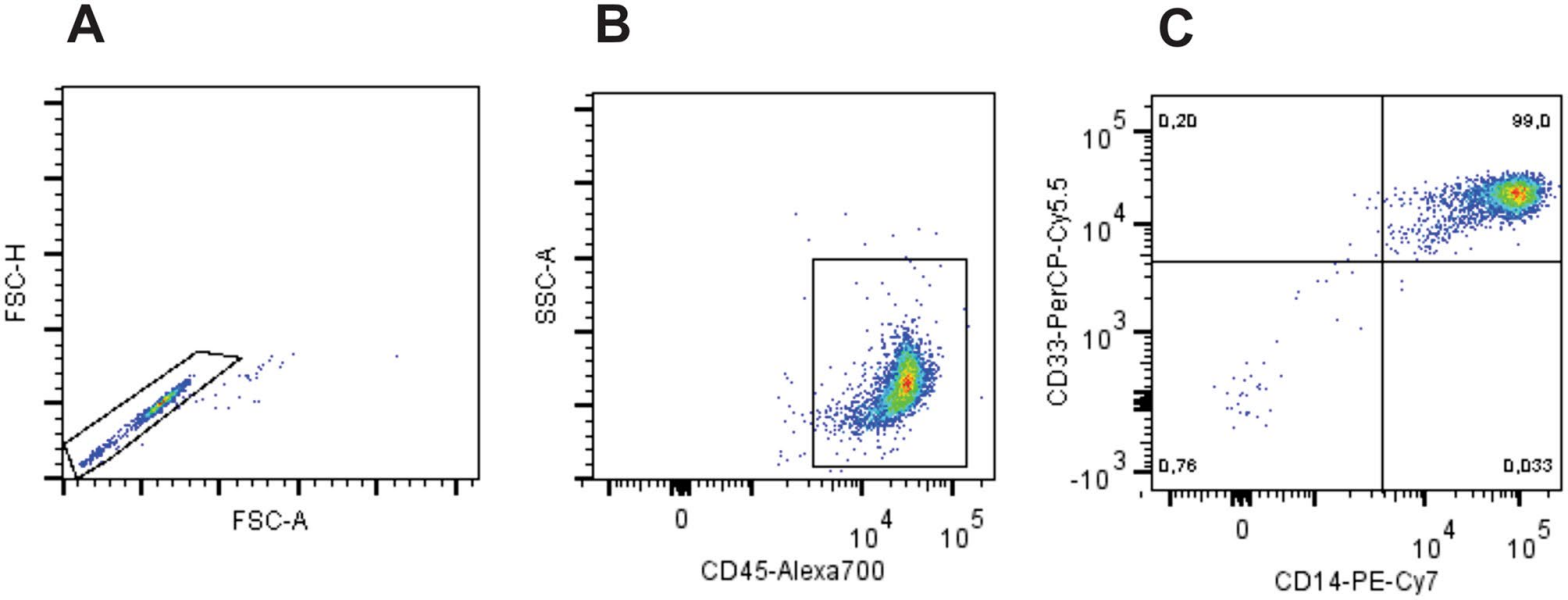
Flow cytometric analysis of CD14 selected monocytes used for dendritic cells differentiation. Flow cytometry dot plot analysis (**A**) Exclusion of doublets, gating on FSC-Area vs FSC-High; (**B**) sequential gate on cells expressing CD45; (**C**) and a final gate displaying 99% purity, almost all cells are identified as monocytes by the co-expression of CD14+ and CD33+.

**Figure 4:**
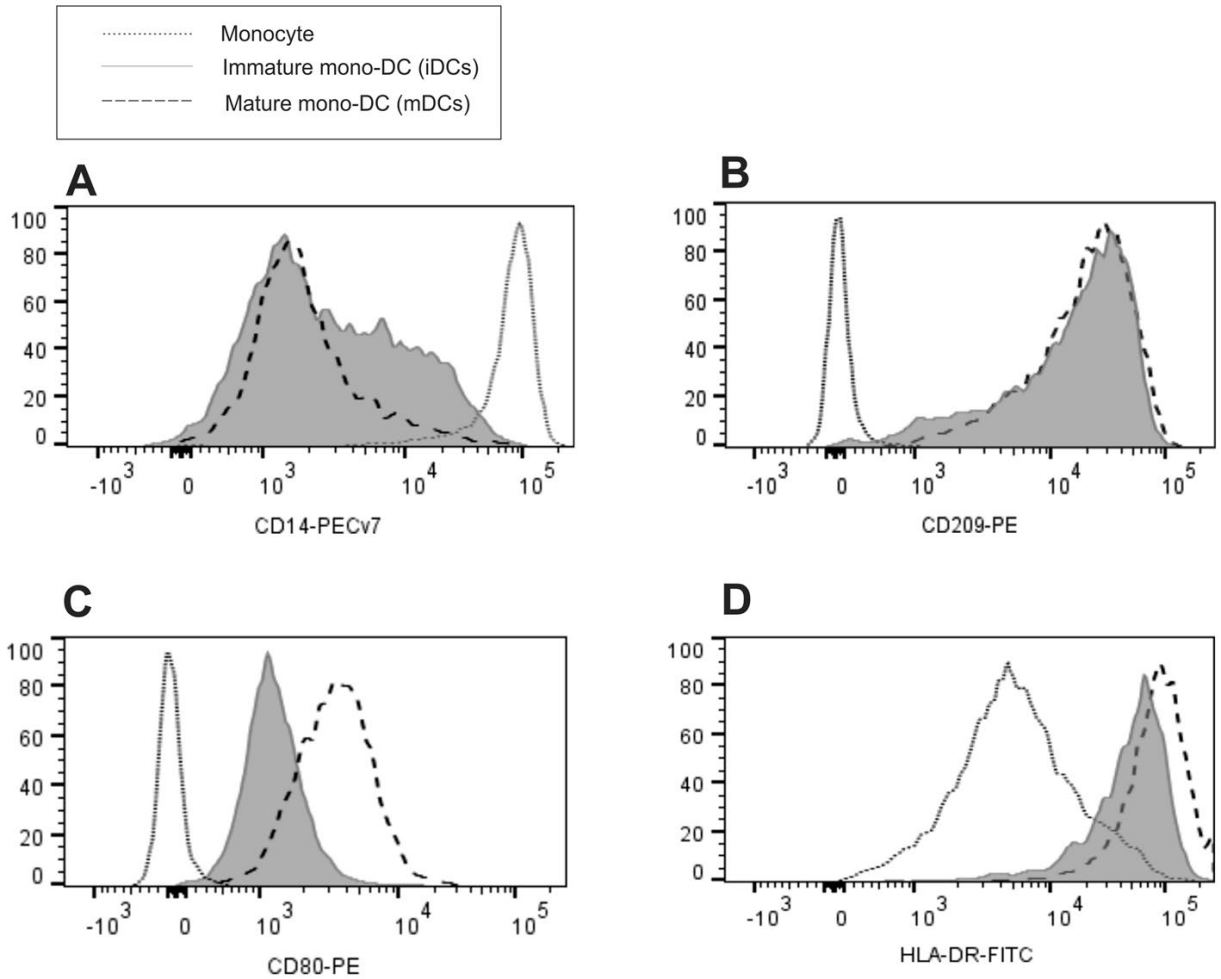
Flow cytometric analysis of monocytes differention into dendritic cells. The differentiation from monocytes was confirmed based on the comparison of CD14, CD209, CD80 and HLA-DR expression between monocytes, iDCs and mDCs, analysis of overlaid histogram by flow cytometry as follows: (**A**) there is decreasing expression of CD14 according to DC status of differentiation and maturation, and increasing expression of (**B**) CD209; (**C**) CD80 and (**D**) HLA-DR. Representative results of n = 8 samples.

Isolation of CD14 + blood monocytes through positive selection yielded 99% purity of monocytes, as demonstrated in Fig. 3. Immature DCs (iDCs) were obtained after cultivation with GM-CSF and IL-4 for 6 days, and mature DCs (mDCs) were obtained following treatment with LPS for additional 24 h. It was possible to observe the decreasing CD14 expression and increased expression of CD209, as expected during differentiation, and increasing levels of CD80 and HLA-DR, characterizing the maturation process (Fig. 4).

**Figure 5:**
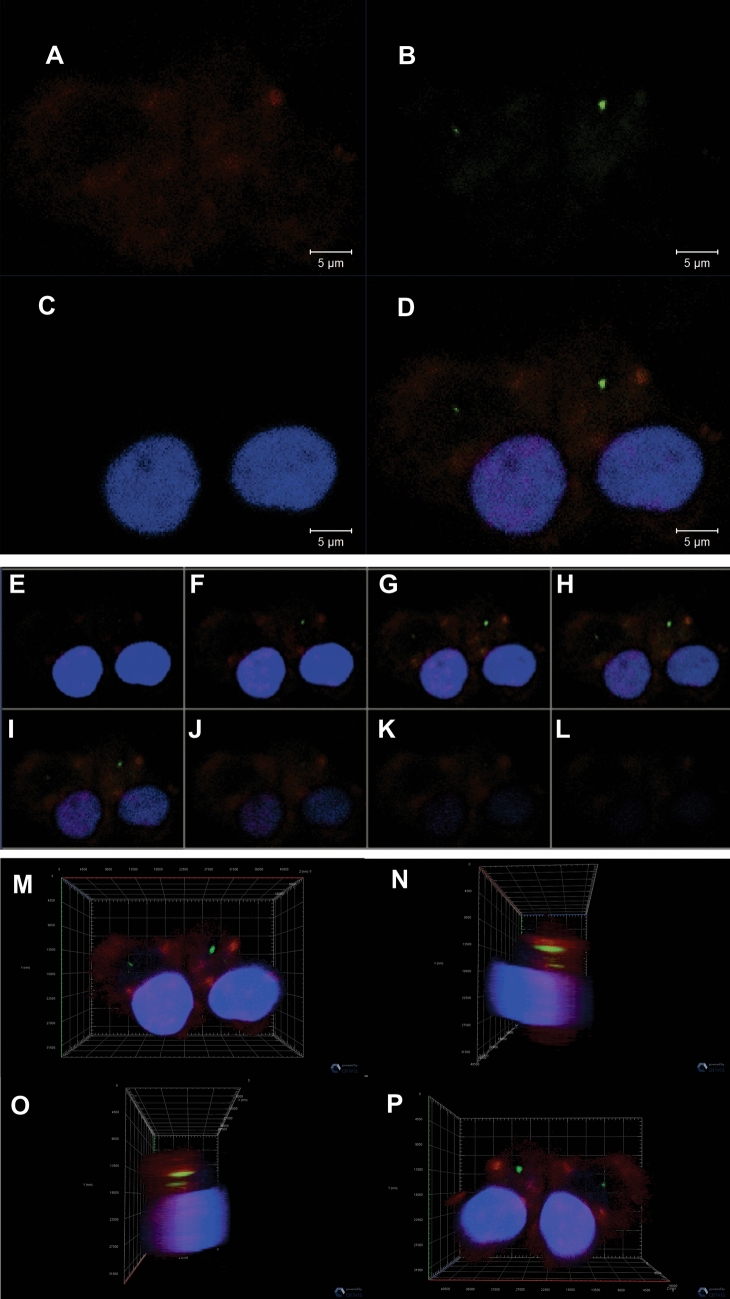
Illustration of EV internalization by mDCs using confocal microscopy. (**A**) cells labeled with CD209 (red), a dendritic cell transmembrane antigen; (**B**) EVs stained with PKH67 (green); (**C**) nuclear staining with DAPI (blue); (**D**) Merge of CD209, DAPI and PKH67; (**E**–**L**) Sequential images using the z-stack tool of confocal microscopy depicting EVs inside the dendritic cells. Three-dimensional images of EVs and mDCs displayed in four angles of 45**°** displaying nuclei (blue), CD209 (red), EVs (green); (**M**) front view of the cells; (**N**) left lateral view of the cells; (**O**) right lateral view of the cells; (**P**) back view of the cells, indicating that the EVs were internalized by mono-DCs.

We then investigated if EVs isolated from cell culture medium could be incorporated by mDCs. The detection of EVs by fluorescence microscopy in live cells is limited by the fast dynamics of fusion events and by microscopy resolution, so in order to investigate these events we used several tools from confocal microscopy. EVs were labeled with PKH67, a green fluorescent dye, and then incubated with mDCs, previously labeled with CD209 (red), a dendritic cell marker (Fig. 5A). Upon addition of EVs to the cell culture, we were able to register that the PKH67-labeled vesicles were taken up by mDCs (Fig. 5B–D). In addition, sequential images using Z-stack (Fig. 5E–L) and three-dimensional images (Fig. 5M–P) corroborated that EVs were incorporated by mDCs.

### EVs interfere with dendritic cells viability, adhesion and migration

**Figure 6:**
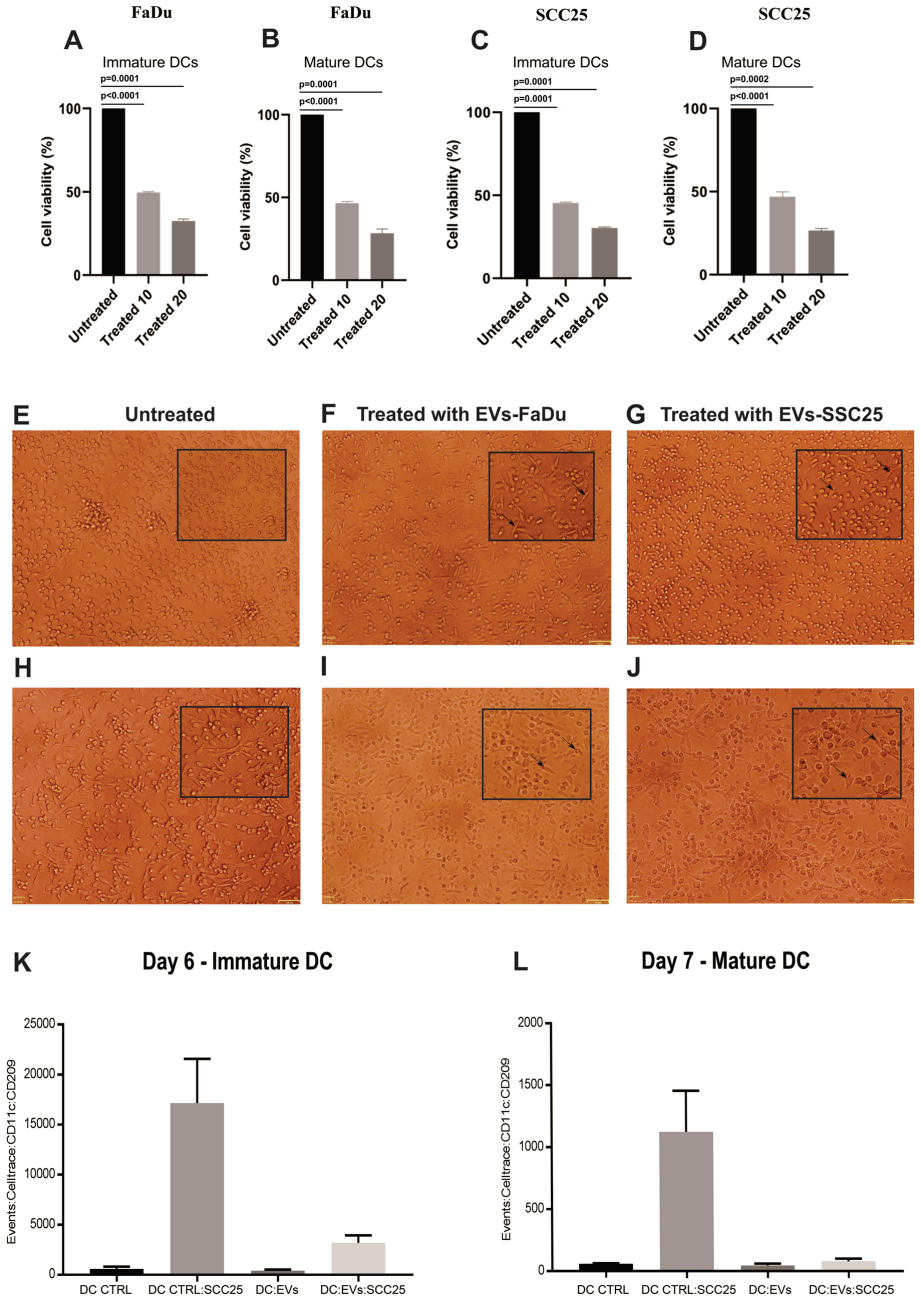
Viability of DCs cultivated in the presence or absence of EVs. Viability of (**A**) iDCs and (**B**) mDCs treated with EVs from FaDu; (**C**) iDCs and (**D**) mDCs treated with EVs from SCC25. Two different concentrations of EVs were used. Representative results of n = 4 samples. Phase contrast microscopy of iDCs and mDCs cultivated in the presence or absence of EVs derived from FaDu or SCC25 cell lines. (**E**) untreated iDCs; (**F**) iDCs treated with FaDu EVs; (**G**) iDCs treated with SCC25 EVs; (**H**) untreated mDCs; (**I**) mDCs treated with FaDu EVs; (**J**) mDCs treated with SCC25 EVs (× 10 magnification). Details from each image were amplified and included as image inserts for better visualization of cells adhered to culture plates. Migration assay analysis. Violet CellTrace-stained iDCs (**K**) and mDCs (**L**) were detected in the lower chamber of the transwells. DC CTRL: untreated iDC or mDC; DC CTRL SCC25: untreated iDC or mDC placed in the transwell with SCC25 in the lower chamber; DCs EVs: iDC or mDC treated with EVs; DC EV SCC25: iDC or mDC treated with EVs placed in the transwell with SCC25 in the lower chamber. Diminished migration was observed for EV-treated iDCs (426 ± 27 cells) and mDCs (45 ± 16 cells) when compared with untreated DCs (controls), and this difference was even more evident when SCC25 cells were placed in the lower chamber: 3190 ± 764 treated iDCs migrated to the lower chamber and 79 ± 23 treated mDCS migrated to the lower chamber, compared with 17,153 ± 4411 untreated iDCs and 1125 ± 132 mDCs migrating in control assays. Representative results of n = 2 samples.

We investigated whether EVs affected the viability of DCs. iDCs (day 6) and mDCs (day 7) were treated with EVs isolated from culture medium. We observed significant reduction in cell viability of mono-DCs cultivated in the presence of EVs derived from both cell lines when compared to the control (untreated) cells (Fig. 6A–D). Viable iDCs showed differences in adhesion when cultivated in the presence of EVs, with higher number of cells attached to the culture well (Fig. 6E–G), and viable mDCs cultivated in the presence of EVs adhered more firmly to the culture dish when compared to the control cells (Fig. 6H–J).

We investigated if the difference in cell adhesion detected in culture dishes could have an impact in DC migration using transwell assays. iDCs and mDCs treated with EVs showed diminished migration when compared with control (untreated) iDCs and mDCs (Fig. 6K,L). These differences were even more evident when SCC25 was placed in the lower chamber, indicating that DCs treated with EVs did not respond to the presence of the cancer cell in the lower chamber (Fig. 6K,L).

### EVs impair differentiation and maturation of mono-DCs

Monocyte-derived iDCs were obtained following CD14 + cells treatment with human recombinant IL-4 and GM-CSF for six days and the maturation signal consisted on treatment of iDCs with LPS for 24 h. Both treatments were carried out either in the presence or absence of EVs derived from FaDu or SCC25 cell lines. Immunophenotyping of monocytes and mono-DCs was based on the detection and expression levels of cell surface markers CD14, CD33, CD80, CD86, CD209 and HLA-DR by flow cytometry.

**Figure 7:**
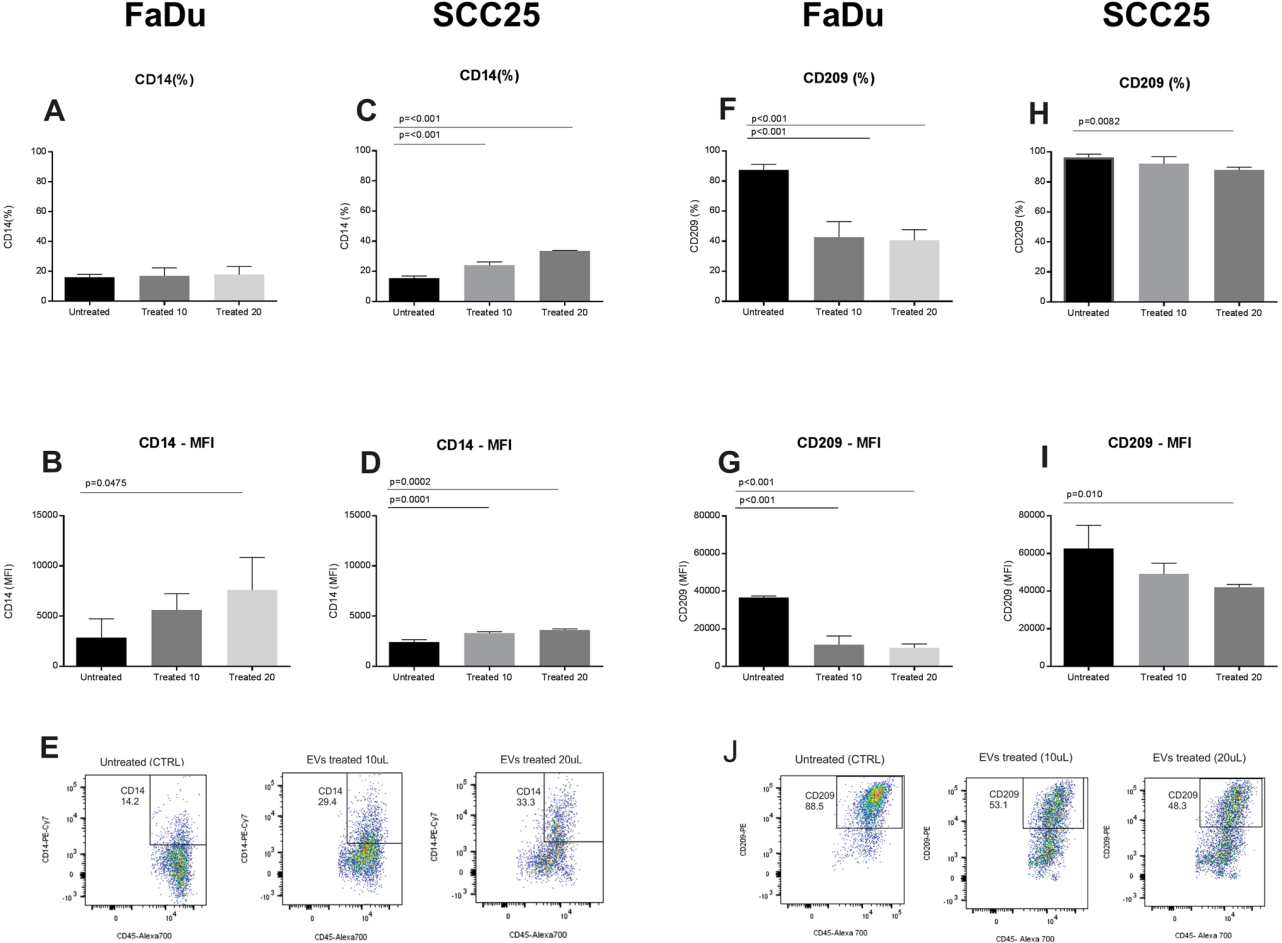
Expression of CD14 and CD209 during dendritic cell differentiation in the presence and absence of EV. CD14 expression following treatment with FaDu cell line EVs: (**A**) There were no significant differences in the percentage of iDCs expressing CD14 between control (16.2% ± 1.8%) and EV treated cells (17.1 ± 5.3% and 17.9 ± 5.5%); (**B**) there were significant differences in the mean intensity of fluorescence (MFI) of CD14 in iDCs between the control (2884 ± 1857) and EV treated samples (5636 ± 1613 and 7605 ± 3265) depending of EV concentration. CD14 expression following treatment with SCC25 cell line EVs: (**C**) There were significant differences in the percentage of iDCs expressing CD14 between the control (15.5 ± 1.3%) and EV treated cells (24.0 ± 2.3% and 33.5 ± 0.3%) depending on EV concentration; (**D**) there were significant differences in CD14 MFI between control (2402 ± 253) and EV treated cells (3275 ± 170 and 3623 + 111) depending on EV concentration; (**E**) Representative dot plots displaying iDCs expressing CD14 on day 6 of culture without treatment (CTRL) and EV-treated cells. CD209 expression following treatment with FaDu cell line EVs: (**F**) There were significant differences in the percentage of iDCs expressing CD209 between control (87.4 ± 3.8%) and EV treated cells (42.8 ± 10.2% and 40.5 ± 7.1%); (**G**) there were differences between untreated iDCs MFI (36,670 ± 802) and treated iDCs (11,543 + 4719 and 9900 ± 2034). CD209 expression following treatment with SCC25 cell line EVs: (**H**) there were significant differences in iDCs expressing CD209, between the control (96.5 ± 2.1%) and treated cells (92 ± 4.8% and 87.9 ± 1.9%), depending on EV concentration; (**I**) there were differences between untreated iDCs MFI (62,514 + 12,407) and treated cells (49,110 ± 5632 and 42,011 ± 1568) (n = 4); (**J**) Representative dot plot graphs displaying iDCs expressing CD209 on day 6 of culture without treatment (CTRL) and EV-treated cells with different concentrations of EVs. Representative results of n = 4 samples per lineage type. The statistical analysis was performed using one way ANOVA with Dunnett correction.

The expression levels of CD209 and CD14 were used as differentiation markers. CD14 is highly expressed in monocytes, and during the differentiation process into mono-DCs the expression of this molecule decreases. On the other hand, CD209 is not expressed in monocytes, and during the differentiation process its expression level gradually increases, being highly expressed in mono-DCs. Our results show that mono-DCs cultivated in the presence of EVs presented higher levels of CD14 when compared with untreated mono-DCs (Fig. 7A–E), while the expression of CD209 was lower in treated mono-DCs when compared with untreated cells (Fig. 7F–J), indicating alterations in the differentiation process.

**Figure 8:**
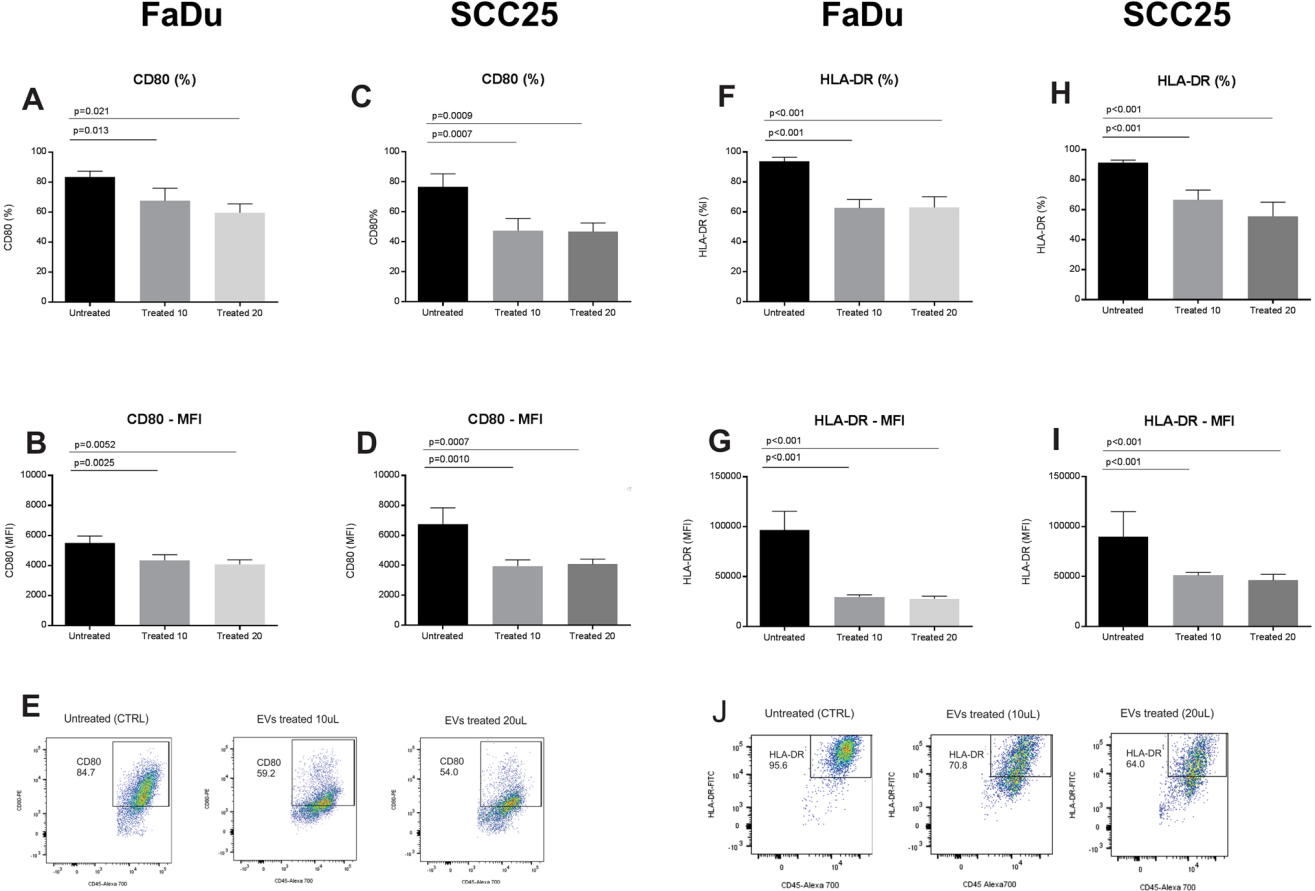
Expression of CD80 and HLA-DR in mDCs both in the presence and absence of EVs derived from the FaDu and SCC25 cell lines. FaDu cell line: (**A**) there was a significant difference in the percentage of mDCs expressing CD80 between the control (83.7 ± 3.7%) and EV-treated cells (67.7 ± 8.4% and 59.6 ± 5.9%); (**B**) there was a significant difference in MFI of CD80 in mDCs between the control (5507 ± 452) and EV-treated cells (4343 ± 374 and 4082 ± 299) depending on EVs concentration. SCC25 cell line: (**C**) there was a significant difference in the percentage of mDCs expressing CD80 between the control (76.5% ± 8.8) and EV-treated cells (47.6 ± 8.0 and 46.8 ± 5.9) at the higher concentration of EVs, (**D)** there was a significant difference in CD80 MFI in mDCs between the control (6735 ± 1106) and EV-treated cells (3943 ± 420 and 4078 ± 336); (**E**) Representative dot plots displaying mDCs expressing CD80 on day 7 of culture without treatment (CTRL) and EV-treated cells. FaDu cell line: (**F**) there were significant differences in the percentage of mDCs expressing HLA-DR between the control (93.6 ± 2.8%) and EV-treated cells (62.6 ± 5.7% and 63 ± 7.1%); (**G**) there were significant differences in HLA-DR MFI between the control (96,646 ± 18,762) and treated cells (29,523 ± 18,762 and 27,865 ± 2527). SCC25 cell line: (**H**) there was a significant difference in the percentage of mDCs expressing HLA-DR between the control (99.3 ± 0.5%) and EV-treated cells (96.6 ± 2.8% and 96.8 + 2.3%); (**I**) there were differences between HLA-DR MFI from control (99,974 + 1,301) and EV-treated cells (66,778 ± 2605 and 59,898 ± 7024); (**J**) Representative dot plots displaying mDCs expressing HLA-DR on day 7 of culture without treatment (CTRL) and EV-treated cells. Representative results of n = 4 samples per lineage type. The statistical analysis was performed using one way ANOVA with Dunnett correction.

The expression levels of the molecules CD80 and HLA-DR were used as markers of cell maturation. The percentage of cells expressing CD80 and the intensity of its expression as determined by MFI were lower following treatment with EVs from FaDu or SCC25 (Fig. 8A–E). Additionally, CD80 was down-regulated at the gene expression level following treatment with EVs. The percentage of cells expressing HLA-DR, on the other hand, was similar both when mono-DCs cells were cultured in the presence or absence of EVs, but the level of HLA-DR expression, as measured by fluorescence intensity, was lower when EVs were present during the maturation signals (Fig. 8F–J). The maturation of DCs is essential for their function in the tumor microenvironment and the differences in surface markers of mono-DCs in the presence of EVs observed here suggest an effect of their content in this process, possibly contributing to the impairment of immune responses.

### Gene expression alterations found in mDCs treated with EVs suggest disrupted immune responses possibly targeted by miRNAs

In order to verify which biological processes were affected following EVs uptake by DCs, gene expression levels of mDCs treated with EVs were compared with mDCs cultivated in the absence of EVs. Major processes that were clearly down-regulated when cells were treated with EVs are listed in Table 1, and include lipopolysaccharide (LPS) response, inflammatory responses, chemokine and chemokine-mediated signaling and cytokine-cytokine receptor interaction. This result suggests that the antigen presenting function of these cells is impaired after the internalization of the EVs. Down-regulated genes when DCs were treated with EVs include *CCL20, FASN*, *IL-1B*, *IL-6*, *IL-12*, *NFκB, NOTCH*, *TMEM97, TGF-β*, and *TNF-α* (Table 1). Additionally, *IL-12*, *TMEM97* and *CCL20* were among the most down-regulated genes in DCs following treatment with EVs (Table 2). This set of genes is highlighted due to their direct role in DC function and to the fact that they are targetted by miRNAs detected within EVs in this study: miR-16, miR-23b, miR- 24, miR-17-5p, miR-21, miR-152, miR-155, miR-181, and miR-212 (Table 3). Within this group of miRNAs, a subset has been previously detected in plasma from HNSCC patients, both in previous reports and in our cohort: miR-17-5p in OPHSCC, miR-21 in OSCC, and miR-16, miR-24, miR-181a circulating in plasma of both OSCC and OPHSCC patients. MiR-23b, which had not been previously described in plasma of HNSCC, was found in this work in plasma of both OSCC and OPSCC patients (Supplementary Table S2).

**Table 1:** Biological processes down regulated in mDCs treated with EVs when compared with untreated mDCs.

GO term	Genes	FDR p-value
GO:0006695: cholesterol biosynthetic process	*TM7SF2, EBP, MSMO1, MVD, CYP51A1, HMGCR, HMGCS1, FDPS, ACLY, FDFT1, INSIG2, SQLE, DHCR7, INSIG1, MVK, IDI1, HSD17B7, NSDHL, DHCR24*	1.44E-15
GO:0006954: inflammatory response	*CXCL1, C3AR1, CCL2, TNF, PTGS2, ADORA2A, AIF1, FPR1, CXCL8, NFKB1, NFKB2, TLR5, TLR8, CCL24, PTGIR, IL23A, REL, CCL20, CCL3L1, CCL3L3, MGLL, IL1B, ZC3H12A, PTX3, NFKBIZ, IL6, GBP5, ADGRE2, OLR1, HCK, CD180, CCL17, RPS6KA5, TNFRSF9, ORM1, TNFAIP6, P2RX7, CCL13, CCR5, CD14, CAMK1D*	2.71E-10
GO:0071294 ~ cellular response to zinc ion	*MT1L, MT1M, MT1A, MT2A, MT1E, MT1B, MT1H, MT1X, MT1G, MT1F*	8.96E-07
GO:0071222: cellular response to lipopolysaccharide	*MEF2C, IL6, TNF, CCL2, CXCL8, NFKB1, TLR5, CD180, SRC, LILRB2, CD36, CCL20, CD80, CCR5, HAMP, ZC3H12A, IL12B, CD14*	1.63E-05
GO:0008285: negative regulation of cell proliferation	*CXCL1, HIST1H2AC, RARRES1, PTGS2, ADORA2A, CXCL8, MEN1, CD9, PTK2B, CCL3L1, CCL3L3, IL1B, NDRG1, ETV3, DHCR24, DFNA5, IL6, KLF11, PIM2, SLC9A3R1, SLIT3, SOD2, OSM, ATF5, NOTCH2, TNFRSF9, RASSF5, BTG2, IRF1, RBPJ, TP53INP1*	6.75E-04
GO:0070098: chemokine-mediated signaling pathway	*CCL24, CXCL1, CCL13, CCL2, CCR5, CCL20, CMKLR1, CCL3L1, PTK2B, CCL3L3, CXCL8, CCL17*	
hsa00100:Steroid biosynthesis	*TM7SF2, EBP, SC5D, MSMO1, SQLE, CYP51A1, DHCR7, HSD17B7, NSDHL, FDFT1, DHCR24*	3.43E-07
hsa04978:Mineral absorption	*ATP1B1, MT1M, MT1A, MT2A, MT1E, MT1B, MT1H, MT1X, MT1G, MT1F*	0.01
hsa04062:Chemokine signaling pathway	*CXCL1, CCL2, NCF1, HCK, CXCL8, NFKB1, SRC, ELMO1, CCL17, CCL24, NRAS, CCL13, CCR5, CCL20, PTK2B, CCL3L1, CCL3L3, GNB4, PAK1, JAK3*	0.05
hsa04060:Cytokine-cytokine receptor interaction	*CXCL1, IL6, IL1R1, TNF, CCL2, TGFBR1, TGFBR2, TNFSF15, CXCL8, CCL17, IL17RB, CCL24, OSM, TNFRSF9, CCL13, IL23A, CCR5, CCL20, CCL3L1, CCL3L3, CSF3R, IL1B, IL12B*	0.04
hsa01212:Fatty acid metabolism	*ACADSB, ACSL1, ELOVL5, FADS1, SCD, HSD17B12, FASN, FADS2, ELOVL6, ACAT2*	0.04

The p value was FDR corrected.

**Table 2:** Ten most differentially expressed genes between mDCs treated with EVs and untreated mDCs.

Gene symbol	Fold change: untreated DCs vs treated DCs	FDR p-value	Gene name
*MUCL1*	− 189.9	8.42E−07	Mucin-like 1
*MMP12*	− 51.96	3.06E−06	Matrix metallopeptidase 12
*MMP1*	− 41.16	1.06E−06	Matrix metallopeptidase 1
*SPINK1*	− 34.75	6.96E−06	Serine peptidase inhibitor, Kazal type 1
*MMP9*	− 14.87	0.0003	Matrix metallopeptidase 9
*COL6A1*	− 11.85	5.94E−06	Collagen. type VI. alpha 1
*TM4SF1*	− 11.4	0.0003	Transmembrane 4 L six family member 1
*ABCB5*	− 10.69	9.69E−05	ATP binding cassette subfamily B member 5
*COL6A2*	− 9.93	4.86E−05	Collagen. type VI. alpha 2
*TGFBI*	− 7.78	1.54E−05	Transforming growth factor beta-induced. 68 kDa
*IL12B*	16.06	5.63E−05	Interleukin 12B
*HMGCS1*	16.27	8.82E−06	3-Hydroxy-3-methylglutaryl-CoA synthase 1 (soluble)
*CDH1*	20.52	0.0002	Cadherin 1. type 1
*HS3ST2*	24.99	5.94E−06	Heparan sulfate (glucosamine) 3-O-sulfotransferase 2
*RARRES1*	27.49	1.97E−05	Retinoic acid receptor responder (tazarotene induced) 1
*TNFAIP6*	30.71	8.88E−05	Tumor necrosis factor alpha-induced protein 6
*IDO1*	63.77	2.29E−06	Indoleamine 2.3-dioxygenase 1
*TMEM97*	72.65	5.94E−06	Transmembrane protein 97
*ORM1*	74.64	8.42E−07	Orosomucoid 1
*CCL20*	91.37	0.0002	chemokine (C–C motif) ligand 20

Negative numbers: up regulation in treated mDCs. The p value was FDR corrected.

**Table 3:** MiRNAs detected in EVs and respective gene targets associated with immune cell function found to be down-regulated in DCs treated with EVs.

miRNAs^#^	Target gene of miRNAs in DCs*	Reported effects on DCs*	Cell type isolated^#^
miR-16-5p	*FASN*^35^	Decreased differentiation^36^	FaDu and SCC25
miR-23b-5p	*NOTCH* and *NF-kB*^37^	Decreased cytokine production^38^	FaDu and SCC25
miR-24-3p	*TNF-a*, *IL-6* and *IL-12*^39^ *NF-kB*^40^	Decreased antigen processing and presentation^41^ Decreased cytokine production^39^	FaDu and SCC25
miR-181a-5p	*TNF-a*, *IL-1B*, and *IL-6*^42^ *TNF-a*^43^	Decreased cytokine production^42^	FaDu and SCC25
miR-17-5p	*TNF-a* and *IL-12*^44^	Decreased maturation^44^	SCC25
miR-21-5p	*TNF-a*^45^ *CCL20*^46^	Decreased migration and cytokine production^47^	SCC25
miR-152-3p	*TMEM97*^48^	Decreased antigen processing and presentation^49^	SCC25
miR-155-5p	*IL-1*^50^	Increased cell death^51^ Decreased maturation^52^	SCC25
miR-212-3p	*TGF-B*^53^	Decreased antigen processing and presentation^29^	SCC25

Target genes reported here were studied in monocytes and/or dendritic cells according to references included in this table.

^#^Reported in this work.

*Reported in this work and literature.

## Discussion

Disruption of DC antigen presentation machinery has been extensively studied and shown to be one of the most significant strategies used by tumor cells to guarantee their survival and cancer progression^54,55^. EVs have been directly implicated in the cross talk between cancer cells and immune cells^29,56–58^.

A previous report on the effect of EVs derived from an oral SCC cell line on monocytes and DCs showed decreased expression of antigen presentation-related components and impaired cell differentiation of DCs^59^. In our study we corroborate these results for another cell line derived from oral SCC (SCC25), and add results for a cell line derived from SCC of the hypopharynx (FaDu). Additionally, using DNA microarrays, we found significant effects on biological processes associated with DC function when mono-DCs where treated with SCC25 or FaDu-derived EVs, including the diminished expression of lipopolysaccharide (LPS) response, inflammatory response, chemokine and chemokine-mediated signaling, and cytokine-cytokine receptor interaction.

Our study identified miRNAs within EVs with essential roles in DC function, a subset of these miRNAs target genes found to be down-regulated in DCs cultivated in the presence of EVs (Table 3). In fact, miRNAs have been shown to contribute to the inibition of immune responses when carried by EVs, with effects on monocyte differentiation into macrophages^36^, production of inflammatory cytokines and co-stimulatory molecules by DCs^38,39^, antigen processing and presentation^41^, MHC II molecules expression^29,49^, DC maturation^44,52^, migration^47^, and increased DC death^51^. Additionally, these miRNAs could potentially cooperate to regulate genes involved in these processes since different miRNAs carried by EVs were shown to regulate the same genes: miR-17-5p, miR-21, miR-24 and miR-181 target *TNF-a*^41,44,45^, miR-17-5p and miR-24 target *IL-12*^41,44^, and miR-155 and miR-181 regulate *IL-1*^42,60^.

Even though the effects observed in DCs cannot not be entirely explained by miRNAs, we showed that EVs were internalized by DCs, and found a set of miRNA gene targets down-regulated in DCs following treatment with EVs. Additionally, these gene targets were associated with specific and essential biological processes, supporting the hypothesis that the transport of miRNAs by EVs is a tumor cell signalling tool with immune modulatory objectives in HNSCC.

It is also noteworthy that we identified a set of common miRNAs between EVs derived from SCC cell lines and plasma of HNSCC patients (Supplementary Table S2), suggesting that these plasma circulating miRNAs are derived from cancer cells and are involved in cell signaling affecting distant body sites or circulating immune cells. In fact, DCs cultivated in the presence of lung cancer patient serum have shown decreased expression of *CD40, CD80, CD86, MHCII, IL1* and *NFκb*, similarly to the results we showed here^61,62^.

Finally, literature reports usually address few patients and often mixed HNSCC subsites. Here we analyzed a cohort of individuals bearing only one of two subsites and evaluated not only disease parameters but also sample quality and limitations of the miRNA detection technique.

In conclusion, we detected miRNAs in EVs derived from HNSCC cell lines and circulating in patients’ plasma, corroborating their potential as cancer biomarkers but also their possible implication in cell signaling, and the usefulness of these cancer cell lines to study EVs and cancer cells signaling. We found differences in gene expression and surface markers of mono-DCs cultivated in the presence of EVs collected from cancer cells culture indicating that EV content, including miRNAs, affect differentiation and maturation processes, essential aspects for the proper function of dendritic cells in the tumor microenvironment.

## Methods

### HNSCC-derived cell lines

The cell lines SCC25, derived from a SCC of the tongue, and FaDu derived from a SCC of the hypopharynx, were used in this study. They were obtained directly from American Type Culture Collection (catalog numbers CRL-1628 and HTB-43). The cell lines were grown in a Dulbecco’s Modified Eagle’s medium/Nutrient Mixture F-12 Ham (DMEM/F12) supplemented with 10% fetal bovine serum in a humidified atmosphere of 5% CO_2_ and 95% air at 37 °C.

### Cell culture and EVs isolation from cell supernatant

Isolation of EVs from cell culture supernatant followed the protocols described by Skog et al. and Mathivanan et al.^63,64^. Briefly, SCC25 and FaDu cell lines were cultivated in five independent T75 flasks until 80–90% confluence and then kept in serum-free medium for 24 h. Following this period the medium was collected (10 mL per flask, total of 50 mL per cell line), and centrifuged for 30 min at 2000×*g* to remove any cells or debris. This medium was then submitted to centrifugation (4 h/52,800×*g*) and ultracentrifugation (4 h/110,000×*g*) (Beckman-Coulter 70Ti rotor). The supernatant was discarded and the EVs pellet was ressuspended in 400 μL of PBS and stored at − 80 °C. For the treatment of mono-DCs we used 10 μL or 20 μL of this EV suspension, based on the work of Wang et al. that used the volume of medium as the reference for treating cells with EVs^65^. The analysis of the integrity and morphologic characteristics of EVs by transmission electron microscopy followed traditional protocols^66^. For microarray analysis, EVs from cell culture supernatant were isolated using Total Exosome Isolation Reagent (from cell culture media) (Invitrogen, Thermo Fisher Scientific, Waltham, Massachusetts, USA). Briefly, 5 mL of the isolation reagent were added to the media and mixed by vortexing. The mixture was left overnight at 4 °C. On the following day, the mixture was centrifuged for 1 h at 10,000×*g* at 4 °C. The supernatant was discarded and pellets were resuspended in 400 μL of PBS.

### EVs characterization by flow cytometry

Immunophenotyping was performed for EVs following current protocols^67^ using the following antibodies: CD9-PE (clone:209306) from R&D systems, CD63-FITC (clone: MEM-259) from USBiological and CD81-FITC (clone:JS64) from Beckman Coulter. For fluorescence background control we used the Pure Flow (Becman Coulter), an unstained sample of EVs and a mix of fluorescent particles with different sizes (0.1–0.9 µM) Megamix-Plus FSC (Biocytex). At least 10,000 events were acquired in Moflo Astrios EQ (Beckman Coulter), using FSC-height was used for size identification, 100 uM nozzle, pressure betwenn 15 and 20%, sample pressure 0.3 PSI higher than sheath pressure and threshold adjusted for 488 SSC. Sample acquisition was initiated only after 5 minutos after equipment stabilization. Mask was used in FSC module with P1 mask on FSC-1 and M2 mask in FSC-2. The data analysis was performed using FlowJo software (BD Biosciences, v.10.6.2).

### EVs size measurement by tunable resistive pulse sensing

Size range of isolated EVs was measured, following current recommendations^67^. With this purpose we chose tunable resistive pulse sensing (TRPS) and the qNano equipment (Izon, Cambridge, MA, USA). Nanopores NP200 and calibration beads CPC200 (at 1:1000 dilution) were used. Isolated EVs were diluted 1:20 in 0.01% Tween-20 in PBS, filtered with Filtropur S 0.2 µm (Sarstedt, Alemamja), and measured in the same conditions as the calibration beads. The measurement conditions for samples and calibration particles were as follows: pore stretch 45.01 mm, voltage 0.56 V and pressure steps at 5 and 10 mbar. The Izon Control Suite 3.4 software was used for data recording and for calculating particle sizes.

### miRNA profiling of EVs content

RNA was extracted from EVs using miRCURY RNA Isolation Kit—Biofluids (Exiqon, Qiagen, Vedbæk, Denmark) following protocols provided by the manufacturer. The extracted RNA was evaluated for quality and concentration using the Nanodrop One Microvolume UV–Vis Spectrophotometer (Thermo Fisher Scientific) and stored at − 80 °C. For total miRNA expression profiling of EVs we used the Affymetrix GeneChip miRNA 4.0 Array (Thermo Fisher Scientific). This microarray allows for the evaluation of 2578 mature human miRNAs (miRBase version 20). Sample preparation, hybridization, staining and washing procedures followed the manufacturer’s instructions FlashTag Biotin HSR RNA Labeling Kit, (Thermo Fisher Scientific). Hybridization signals were detected using the GeneChip Scanner 3000 7G (Thermo Fisher Scientific) with Affymetrix GeneChip Command Console Software parameters recommended by the manufacturer. A probeset was considered to be expressed if 50% of the samples in the dataset have DABG (Detected Above Background) values below the DABG threshold (the default DABG was set to 0.05). Array data quality analysis, data summarization and normalization (RMA + DABG) were carried out with the Transcriptome Analysis Console software v. 4.0.1 (Thermo Fisher Scientific).

### Immunophenotyping of monocytes and monocyte-derived dendritic cells during differentiation and maturation processes induced in vitro

Peripheral blood was obtained from healthy volunteers. All volunteers signed a written informed consent after the study was approved by Hospital Israelita Albert Einstein Ethics Committee (protocol number CAEE: 86306218.2.0000.0071). Peripheral blood was diluted 1∶3 with PBS and the suspension was transferred to a 15 mL conical tube containing 5 mL of Ficoll-Paque 1.077 density (GE Healthcare, Chicago, Illinois, USA) and centrifuged (30 min/500×*g*) without brake at 22 °C. The cells from the interface were collected, suspended in cell culture media X-vivo 15 (Lonza, Basel, Switzerland) and centrifuged again (5 min/500×*g*). Monocytes were separated using CD14 positive antibody attached to beads, and selected by magnectic columns (Myltenyi Biotec, Bergisch Gladbach, Germany). The selected CD14 cell population was dispensed into six-well plates containing X-vivo 15 medium (Lonza, Basel, Switzerland) supplemented with antibiotic-antimycotics (Gibco, Thermo Fisher Scientific). In order to generate immature monocyte-derived dendritic cells (iDCs), the cells were cultured in the presence of human recombinant IL-4 (20 ng/mL) and GM-CSF (50 ng/mL) (both from R&D Systems, Minneapolis, MN) for six days. Mature mono-DCs (mDCs) were obtained after iDC stimulation with LPS 100 ng/mL for 24 h (Sigma Aldrich)^68^. Cells were cultivated the in presence or absence of EVs. The EVs were added on day 0, the first day of monocytes culture, and during dendritic cells differentiation (iDCs), culture day 4^69^. The EVs used to treat monocytes and iDCs were collected from the culture supernatant of FaDu and SCC25 cell lines and added in two different concentrations (see “Cell culture and EVs isolation from cell supernatant”) to the same medium in which monocytes and mono-DCs were being cultured (X-vivo 15). The EVs were filtrated using a seringe filter of 0.22 µm, before addtion to the cultures.

Immunophenotyping was performed for monocytes isolation, and during mono-DCs differentiation (iDCs) and maturation (mDCs), with the following antibodies: CD33-APC (clone:P67.6) and HLA-DR-PerCP-Cy5.5 (clone:G46.6), from BD Biosciences, CD209-FITC (clone:DCN46), CD8-APC-Cy7 (clone:SK1), CD80-PE (Clone:L307.4), CD86-PE (Clone:2331-FUN-1), CD14-PerCP-Cy5.5 (clone:M5E2). Fluorescence minus one (FMO) was used as a fluorescence background control. At least 10,000 events were acquired using FACS LSRII FORTESSA (BD Biosciences). The data analysis was performed using FlowJo software v.10.6.2 (BD Biosciences, https://www.flowjo.com). The statistical analysis was performed using the GraphPad Prism software v.7.02 (GraphPad Software, San Diego, CA, https://www.graphpad.com/scientific-software/prism).

### Detection of incorporation of EVs by mature monocyte-derived dendritic cells

EVs collected from the culture supernatant of FaDu and SCC25 cell lines were diluted in two different concentrations using the same medium in which monocytes and mono-DCs were being cultured (X-vivo 15). Before using the EVs they were filtered usinge a serynge filter 0.22 µm. Cell line-derived EVs were incubated with the membrane dye PKH67 (green fluorescent cell linker, Sigma, St. Louis, Missouri, USA) according to manufacturer’s instructions. Free dye was eliminated by adding PBS to the labeled EVs, centrifuging the mixture at (1 h/150,000×*g*) and then carefully discarding the supernatant. The PKH67-stained EVs were incubated with mDCs for 24 h. Cells were centrifuged at (10 min/500×*g*), the supernatant was discarded and this procedure was repeated twice with PBS to remove the non-internalized remaining EVs. Next, cells were transferred to a glass slide by cytospin centrifugation, and fixed with 2% formaldehyde for 1 h. Cells were further permeabilized with Triton-X100 0.05% for 30 min and incubated in blocking solution (1% BSA in PBS) and mouse anti-human CD209 (1:200, BD) primary antibody overnight at room temperature. Next, cells were washed twice and incubated with secondary anti-mouse antibody coupled with Alexa-568 (1:100, Invitrogen) in blocking solution for 1 h. After final wash in blocking solution, cells were rinsed three times with PBS and mounted in 1:10 PBS diluted Fluoromount-G with DAPI (eBiosciences, San Diego, California, USA). Cells were analyzed by confocal microscopy (Zeiss 710 Confocal Microscope, Zeiss, Germany) with specific staining for CD209 (red), EVs (green), and nuclei (blue), dysplayed individually and overlapped. In addition, the Z-stack tool was used for sequential photos with diferent deepness, and a 3D photo was captured in order to show the localization of EVs inside the mDC.

### Cell viability analysis of mono-DCs treated with EVs

The evaluation of the viability of mono-DCs cultivated in the presence and absence of EVs was performed by trypan blue staining and cell count in Neubauer chamber. iDCs (day 6) and mDCs (day7) cultivated in the presence or absence of EVs derived from FaDu or SCC25 cell lines were observed using phase contrast microscopy (FSX100, Olympus, Tokyo, Japan).

### Migration assay

Migration of iDCs (day 6) and mDCs (day 7) cultivated in the presence or absence of EVs derived SCC25 was evaluated using a transwell assay. iDCs or mDCs were collected and centrifuged (5 min/500×*g*) and stained using CellTrace Violet BV421 (Thermo Fisher Scientific) for 15 min at 37 °C, and then washed with PBS. Following the staining, cells were seeded on the upper chamber of a transwell (5.0 µM pores) in 24 well plates. DCs migration was induced using SCC25 cells cultivation in the lower chamber of the transwell, while for controls only cell medium was used in the lower chamber of the transwell. After 24 h the percentage of cells that migrated was evaluated by flow cytometry detecting CellTrace Violet stained DCs (iDCs and mDCs) and the antibodies CD11c- PE-CF594 (clone:B-ly6) from BD—Pharmingen and CD209—PerCP-Cy5.5 (clone:DCN46) from BD—Horizon. The events were acquired using FACS LSRII FORTESSA (BD Biosciences). The data analysis was performed using FlowJo software v.10.6.2 (BD Biosciences, https://www.flowjo.com). The statistical analysis was performed using the GraphPad Prism software v. 7.02 (GraphPad Software, San Diego, CA, https://www.graphpad.com/scientific-software/prism).

### Gene expression profiling of monocyte-derived dendritic cells

RNA was extracted from mono-DC samples using the RNEasy Mini Kit (Qiagen, Hilden, Germany**)**. Briefly, cells were washed with ice-cold PBS, lysis buffer was added directly to cells and the protocol was carried out according to the manufacturer’s instructions. RNA integrity was assessed using the Agilent 2100 Bioanalyzer and the RNA 6000 Nano Kit (Agilent Technologies, Santa Clara, CA, USA) and total RNA was stored at − 80 °C until use. Gene expression was analyzed using DNA microarrays Clariom S Arrays (Thermo Fisher Scientific) following the protocols provided by the manufacturer. Hybridization signals were detected using the GeneChip Scanner 3000 7G (Thermo Fisher Scientific) with Affymetrix GeneChip Command Console Software parameters recommended by the manufacturer. A probeset was considered to be expressed if 50% of the samples in the dataset had DABG (Detected Above Background) values below the DABG threshold (the default DABG was set to 0.05). Array data quality analysis, data summarization and normalization (RMA + DABG) and statistical tests for differential expression (eBayes) were carried out with the Transcriptome Analysis Console software (TAC 4.0.1) (cut-off for significance FDR corrected p-value < 0.05). Gene expression levels are presented in fold-changes. Gene Ontology (GO) term enrichment analysis was carried out using DAVID Bioinformatics Resources 6.8 and results were considered statistically significant for p-value < 0.05 after Benjamini–Hochberg False-Discovery Rate (FDR) correction.

### Literature search for plasma miRNAs in HNSCC and associated with DC function

We used PubMed Central (PMC) for literature search and only articles published in English and containing the terms “Head and neck cancer”, “HNSCC” and “plasma” were selected for the correlation of miRNAs found within EVs and HNSCC patients’ plasma (Supplementary Table S2). Similarly, an association between miRNAs and processes associated with DC function found to be deregulated in this work was investigated using the search terms “dendritic cell function”, “differentiation”, “maturation”, “migration” and “dendritic cell”.

### Detection of miRNAs in plasma of HNSCC patients

In order to evaluate if miRNAS found in EVs represented miRNAs circulating in plasma from HNSCC, we evaluated plasma miRNAs in a cohort of patients. Since literature reports usually address few patients and often mix HNSCC subsites, and techniques for detection of miRNAs are very diverse, we decided to analyze a cohort of individuals bearing HNSCC from which we could evaluate disease parameters as well as sample quality and limitations of the miRNA detection technique. The cohort comprised 15 patients with confirmed anatomopathological diagnosis of oropharyngeal squamous cell carcinoma (OPHSCC) and 30 oral cavity squamous cell carcinoma (OSCC) treated at Hospital Heliopolis and Hospital das Clinicas, Sao Paulo, Brazil. This study was approved by the Hospital Heliopolis Ethics Committee, Hospital das Clinicas Ethics Committee and by the National Commission of Ethics in Research (CONEP) (protocol number CAEE: 22983313.3.0000.0071). All individuals recruited for this study provided written informed consent and the research has been performed in accordance with the Declaration of Helsinki. Tumors were staged according to the Union for International Cancer Control (UICC)/American Joint Committee on Cancer (AJCC) staging classification system for head and neck SCC (8th edition). All patients were older than 50 years at the time of sample collection, heavy smokers and had a history of chronic alcohol use. Clinical and pathological data of the individuals participating in this study are summarized in Supplementary Table S2. Peripheral blood was collected in EDTA tubes for plasma separation. The levels of free hemoglobin in the plasma samples were evaluated by spectrophotometry analysis, through the spectrophotometer NanoVue Spectrophotometer (GE Healthcare). The absorbance peaks at 414 nm are indicative of free hemoglobin^70^. The evaluation of the expression levels of miRNAs in different levels of hemolysis was performed with the aid of a hemolysis curve that simulated possible disruptions of red cells in the plasma. For the hemolysis curve, we use different concentrations of red cells in the plasma (0.08%, 0.16%, 0.33% and 0.66% of red blood cell volume/plasma volume) (Supplementary Fig. S1). We considered values above 0.2 of absorbance as a marker of hemolysis since above this value changes in the detection of miRNAs have been reported^71^. RNA extraction from 200 μL of plasma followed the same protocol for miRNA expression profiling of EV content using the miRCURY RNA Isolation Kit—Biofluids (Exiqon).  MiRNA profiling in plasma samples was carried out with Serum/Plasma Focus microRNA PCR panel (Exiqon). For cDNA synthesis we used miRCURY LNA Universal RT cDNA Synthesis Kit (Exiqon) and PCR reactions were carried out according to the protocol for the miRCURY LNA Universal RT microRNA PCR System (Exiqon). The amplification was performed in 7500 Real-Time PCR System (Thermo Fisher Scientific) in 96 well plates. The amplification curves were analyzed using the 7500 software (Thermo Fisher Scientific), both for determination of Ct (Cycle Threshold) and for melting curve analysis. According to manufacturer's instructions, we considered miRNAs as “detected” when Ct ≤ 37.

## Supplementary Information

Supplementary Figure S1.

Supplementary Table S1.

Supplementary Table S2.

Supplementary Table S3.
