# Supplementary material for: Extracellular vesicles cargo from head and neck cancer cell lines disrupt dendritic cells function and match plasma microRNAs
Source: Sci Rep. 2021 Sep 17;11:18534. doi: 10.1038/s41598-021-97753-y (PMC8448882; doi:10.1038/s41598-021-97753-y)
Supplement: Supplementary file 1 — Supplementary Figure S1. [file 41598_2021_97753_MOESM1_ESM.docx]

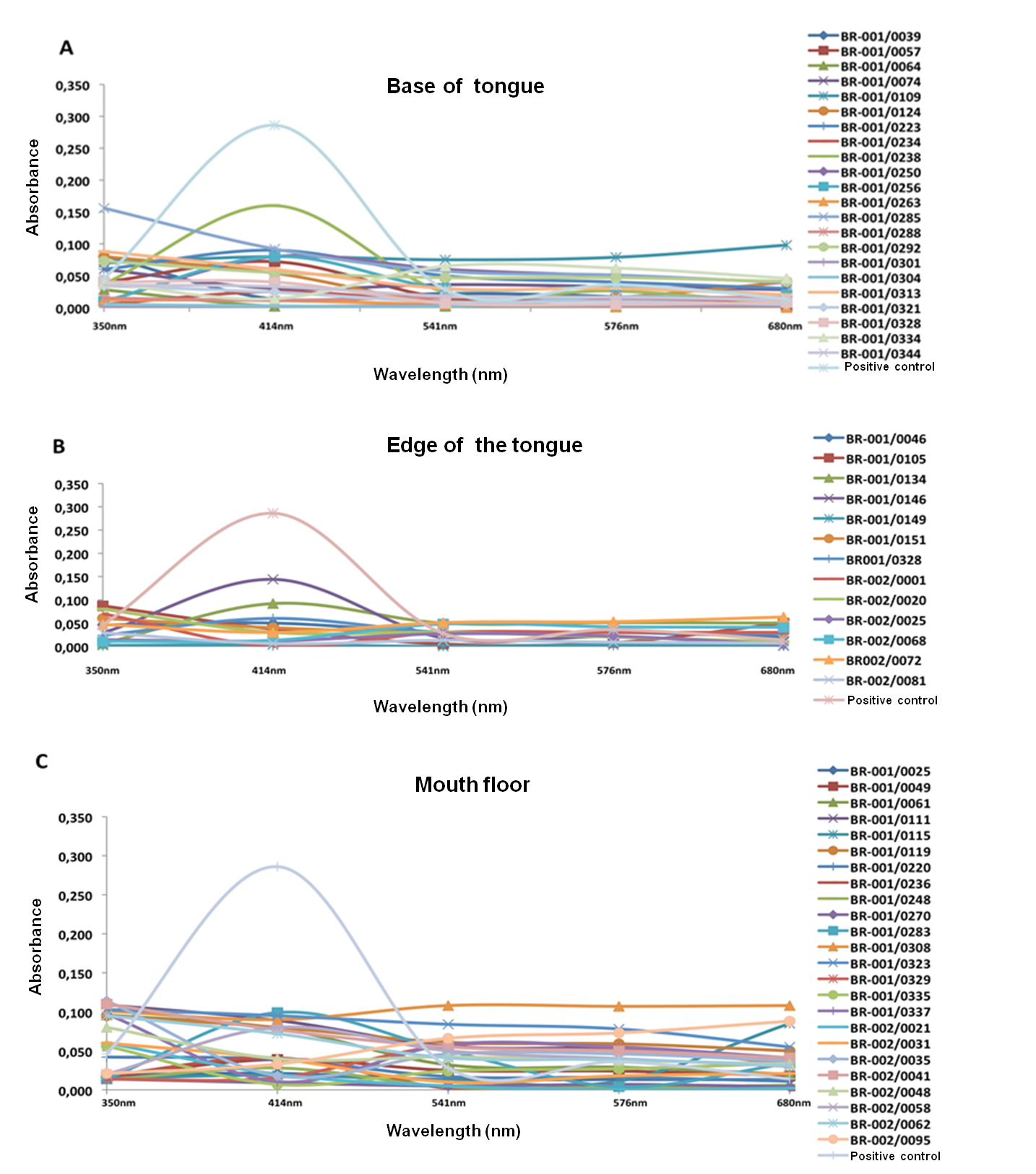


**Supplementary Fig. S1:** Analysis of hemolysis contamination in plasma samples. Absorbance analysis using spectrophotometry as an indicator of plasma hemolysis in plasma. Absorbance values above 0.2 at 414nm indicate hemolysis. (A) base of tongue tumor samples (OPHSCC) (B) edge of tongue tumor samples (OSCC) (C) mouth floor tumors (OSCC). Positive control=plasma samples with red blood cells (0.08%).
