# Supplementary material for: Extracellular vesicles cargo from head and neck cancer cell lines disrupt dendritic cells function and match plasma microRNAs
Source: Sci Rep. 2021 Sep 17;11:18534. doi: 10.1038/s41598-021-97753-y (PMC8448882; doi:10.1038/s41598-021-97753-y)
Supplement: Supplementary file 2 — Supplementary Table S1. [file 41598_2021_97753_MOESM2_ESM.docx]

| miRNA detected in SCC25 EVs | SCC25 Avg (log2) | SCC25 Standard Deviation | miRNA detected in FaDu EVs | FADU Avg (log2) | FADU Standard Deviation |
| --- | --- | --- | --- | --- | --- |
| hsa-miR-103a-3p | 10,75 | 0,1 | **hsa-let-7a-5p** | 9,65 | 0,07 |
| hsa-miR-106b-5p | 7,39 | 0,07 | **hsa-let-7b-5p** | 12,02 | 0,02 |
| hsa-miR-107 | 10 | 0,05 | **hsa-let-7c-5p** | 10,87 | 0,14 |
| hsa-miR-125a-5p | 8,07 | 0,01 | **hsa-let-7d-3p** | 1,14 | 1 |
| hsa-miR-125b-5p | 7,22 | 0,22 | **hsa-let-7d-5p** | 8,42 | 0,03 |
| hsa-miR-126-3p | 2,75 | 0,17 | **hsa-let-7e-5p** | 7,63 | 0,13 |
| hsa-miR-132-3p | 6,7 | 0,34 | **hsa-let-7f-5p** | 4,12 | 0,78 |
| hsa-miR-136-3p | 1,48 | 0,25 | **hsa-let-7g-5p** | 2,72 | 0,61 |
| hsa-miR-138-5p | 4,15 | 0,43 | **hsa-let-7i-5p** | 4,93 | 0,21 |
| hsa-miR-139-5p | 2,49 | 0,81 | **hsa-miR-100-5p** | 3,02 | 0,29 |
| hsa-miR-140-3p | 3,54 | 0,48 | **hsa-miR-103a-3p** | 5,88 | 0,25 |
| hsa-miR-141-3p | 2,67 | 0,79 | **hsa-miR-106a-5p** | 5,12 | 0,19 |
| hsa-miR-151a-3p | 7,48 | 0,02 | **hsa-miR-106b-5p** | 3,86 | 0,1 |
| hsa-miR-151a-5p | 9,33 | 0,24 | **hsa-miR-107** | 5,44 | 0,07 |
| hsa-miR-151b | 4,88 | 0,44 | **hsa-miR-125a-5p** | 4,94 | 0,31 |
| hsa-miR-152-3p | 6,74 | 0,01 | **hsa-miR-125b-5p** | 2,57 | 0,16 |
| hsa-miR-155-5p | 7,76 | 0,07 | **hsa-miR-129-5p** | 4,12 | 0,01 |
| hsa-miR-16-5p | 11,19 | 0,52 | **hsa-miR-130b-3p** | 2,28 | 1,06 |
| hsa-miR-17-5p | 10,52 | 0,02 | **hsa-miR-135a-3p** | 3,34 | 0,33 |
| hsa-miR-181a-5p | 8,41 | 0,22 | **hsa-miR-150-3p** | 6,38 | 0,29 |
| hsa-miR-181b-5p | 8,13 | 0,14 | **hsa-miR-151a-3p** | 4,08 | 0,12 |
| hsa-miR-181d-5p | 3,17 | 0,09 | **hsa-miR-151a-5p** | 5,6 | 0,08 |
| hsa-miR-187-3p | 1,64 | 0,34 | **hsa-miR-15a-5p** | 2,77 | 0,28 |
| hsa-miR-18a-5p | 6,18 | 0,08 | **hsa-miR-15b-5p** | 4,28 | 0,03 |
| hsa-miR-193a-5p | 7,05 | 0,09 | **hsa-miR-16-5p** | 6,21 | 0,18 |
| hsa-miR-193b-3p | 8,29 | 0,17 | **hsa-miR-17-5p** | 5,56 | 0,15 |
| hsa-miR-194-5p | 3,73 | 0,4 | **hsa-miR-181a-5p** | 2,39 | 0 |
| hsa-miR-200a-3p | 4,24 | 0,27 | **hsa-miR-181b-5p** | 2,34 | 0,45 |
| hsa-miR-203a | 5,08 | 0,6 | **hsa-miR-182-5p** | 3,98 | 0,22 |
| hsa-miR-21-5p | 3,68 | 2,05 | **hsa-miR-185-5p** | 3,24 | 0,54 |
| hsa-miR-210-3p | 4,8 | 0,57 | **hsa-miR-191-5p** | 6,29 | 0,14 |
| hsa-miR-212-3p | 2,52 | 0,1 | **hsa-miR-193a-5p** | 4,34 | 0,33 |
| hsa-miR-22-3p | 7,38 | 0,1 | **hsa-miR-193b-3p** | 4,44 | 0,4 |
| hsa-miR-224-5p | 3,68 | 0,16 | **hsa-miR-193b-5p** | 4,47 | 0,27 |
| hsa-miR-23b-3p | 12,3 | 0,03 | **hsa-miR-196a-5p** | 2,95 | 0,04 |
| hsa-miR-23b-5p | 3,02 | 0,06 | **hsa-miR-200b-3p** | 2,41 | 0,55 |
| hsa-miR-24-3p | 12,77 | 0,07 | **hsa-miR-200b-5p** | 1,76 | 0,5 |
| hsa-miR-26a-5p | 10,5 | 0,05 | **hsa-miR-200c-3p** | 9,91 | 0,09 |
| hsa-miR-27a-3p | 7,82 | 0,45 | **hsa-miR-205-5p** | 7 | 0,01 |
| hsa-miR-27b-3p | 9,54 | 0,2 | **hsa-miR-20a-5p** | 4,67 | 0,27 |
| hsa-miR-27b-5p | 4,04 | 0,21 | **hsa-miR-210-3p** | 4,18 | 0,37 |
| hsa-miR-28-5p | 5,02 | 0,04 | **hsa-miR-211-3p** | 3,19 | 0,34 |
| hsa-miR-30a-3p | 2,7 | 0,58 | **hsa-miR-22-3p** | 2,05 | 0,78 |
| hsa-miR-30c-5p | 6,22 | 0,13 | **hsa-miR-221-3p** | 5,08 | 0,14 |
| hsa-miR-31-5p | 10,76 | 0,04 | **hsa-miR-222-3p** | 5,25 | 0,01 |
| hsa-miR-3135b | 4,89 | 0,38 | **hsa-miR-23a-3p** | 7,96 | 0,03 |
| hsa-miR-320d | 9,98 | 0,01 | **hsa-miR-23b-3p** | 5,75 | 0,01 |
| hsa-miR-320e | 7,19 | 0,1 | **hsa-miR-24-3p** | 6,73 | 0,08 |
| hsa-miR-324-5p | 5,86 | 0,29 | **hsa-miR-25-3p** | 4,18 | 0,68 |
| hsa-miR-342-3p | 6,66 | 0,1 | **hsa-miR-25-5p** | 6,95 | 0,38 |
| hsa-miR-342-5p | 2,14 | 0,04 | **hsa-miR-26a-5p** | 5,48 | 0,02 |
| hsa-miR-34c-3p | 1,8 | 0,15 | **hsa-miR-27a-3p** | 2,9 | 0,48 |
| hsa-miR-365a-5p | 3,93 | 0,38 | **hsa-miR-27b-3p** | 2,25 | 0,49 |
| hsa-miR-371b-5p | 5,27 | 0,27 | **hsa-miR-29a-3p** | 2,36 | 0,57 |
| hsa-miR-378c | 4,2 | 0,15 | **hsa-miR-30a-5p** | 1,86 | 0,15 |
| hsa-miR-425-5p | 5,7 | 0,22 | **hsa-miR-30c-5p** | 3,14 | 0,78 |
| hsa-miR-486-5p | 6,15 | 0,05 | **hsa-miR-30d-5p** | 3,52 | 0,26 |
| hsa-miR-498 | 3,92 | 0,25 | **hsa-miR-31-5p** | 6,66 | 0,31 |
| hsa-miR-503-5p | 4,56 | 0,29 | **hsa-miR-320a** | 8,12 | 0,05 |
| hsa-miR-505-5p | 2,83 | 0,13 | **hsa-mir-320a** | 4,57 | 0,29 |
| hsa-miR-574-3p | 5,93 | 0,31 | **hsa-miR-320b** | 7,99 | 0,09 |
| hsa-miR-584-5p | 4,35 | 0,28 | **hsa-miR-320c** | 7,95 | 0,02 |
| hsa-miR-652-3p | 4 | 0,02 | **hsa-miR-320d** | 6,82 | 0,1 |
| hsa-miR-877-5p | 7,15 | 0,21 | **hsa-mir-320e** | 6,04 | 0,05 |
| hsa-miR-92b-3p | 3,49 | 0,05 | **hsa-miR-320e** | 4,47 | 0,5 |
| hsa-miR-93-5p | 9,31 | 0,21 | **hsa-mir-361** | 2,74 | 0,27 |
| hsa-miR-99b-3p | 2,95 | 1,09 | **hsa-miR-361-5p** | 3,84 | 0,19 |
| hsa-miR-99b-5p | 8,95 | 0,23 | **hsa-miR-371b-5p** | 6,91 | 1,13 |
|  |  |  | **hsa-miR-423-5p** | 5,07 | 0,26 |
|  |  |  | **hsa-miR-425-5p** | 2,45 | 0,32 |
|  |  |  | **hsa-miR-455-3p** | 5,45 | 0,45 |
|  |  |  | **hsa-miR-485-5p** | 2,66 | 0,38 |
|  |  |  | **hsa-miR-498** | 4,08 | 0,29 |
|  |  |  | **hsa-miR-505-5p** | 1,9 | 0 |
|  |  |  | **hsa-miR-510-5p** | 1,86 | 0,8 |
|  |  |  | **hsa-miR-564** | 2,91 | 0 |
|  |  |  | **hsa-miR-572** | 8,1 | 0,19 |
|  |  |  | **hsa-miR-584-5p** | 2,42 | 0,41 |
|  |  |  | **hsa-miR-602** | 7,21 | 0,2 |
|  |  |  | **hsa-miR-654-5p** | 4,75 | 0,48 |
|  |  |  | **hsa-miR-663a** | 12,28 | 0,05 |
|  |  |  | **hsa-miR-664a-5p** | 1,77 | 1 |
|  |  |  | **hsa-miR-664b-5p** | 7,06 | 0,08 |
|  |  |  | **hsa-miR-665** | 4,81 | 0,21 |
|  |  |  | **hsa-miR-675-5p** | 4,88 | 0,84 |
|  |  |  | **hsa-miR-744-5p** | 7,36 | 0,39 |
|  |  |  | **hsa-miR-760** | 3,59 | 0,03 |
|  |  |  | **hsa-mir-885** | 7,75 | 0,03 |
|  |  |  | **hsa-miR-885-3p** | 7,02 | 0,3 |
|  |  |  | **hsa-miR-920** | 3,21 | 0,38 |
|  |  |  | **hsa-miR-92a-3p** | 8,07 | 0,02 |
|  |  |  | **hsa-miR-92b-5p** | 9,68 | 0,24 |
|  |  |  | **hsa-miR-93-5p** | 5,52 | 0,31 |
|  |  |  | **hsa-miR-936** | 2,94 | 0,14 |
|  |  |  | **hsa-miR-943** | 2,47 | 0,74 |
|  |  |  | **hsa-miR-99b-5p** | 4,92 | 0,29 |

**Supplementary Table S1:** MicroRNAs detected in EVs FaDu and SCC25 using Affymetrix GeneChip miRNA 4.0 Array. Avg (log2): log2 of average of signals detected above background
