# Supplementary material for: Extracellular vesicles cargo from head and neck cancer cell lines disrupt dendritic cells function and match plasma microRNAs
Source: Sci Rep. 2021 Sep 17;11:18534. doi: 10.1038/s41598-021-97753-y (PMC8448882; doi:10.1038/s41598-021-97753-y)
Supplement: Supplementary file 3 — Supplementary Table S2. [file 41598_2021_97753_MOESM3_ESM.docx]

| **This study** | | | | | | | **Literature** | | | |
| --- | --- | --- | --- | --- | --- | --- | --- | --- | --- | --- |
| **MiRNA** | **EVs FaDu** | **EVs SCC25** | **Both EVs** | **OPHSCC patients** | **OSCC patients** | **Both tumor subtypes** | **Family of miRNA** | **Biofluid** | **Subtype** | **Reference** |
| Hsa-mir-181b-5p | Yes | Yes | Yes | - | - | - | Family | Plasma | OSCC | ^1^ |
| Hsa-mir-584-5p | Yes | Yes | Yes | - | - | - | - | Serum | NPC | ^2^ |
| Hsa-mir-30c-5p | Yes | Yes | Yes | Yes | Yes | Yes | - | Plasma/Serum | NPC/SCC | ^3-5^ |
| Hsa-mir-26a-5p | Yes | Yes | Yes | Yes | Yes | Yes | - | Plasma/Serum | OSCC/NPC | ^6-8^ |
| Hsa-mir-17-5p | Yes | Yes | Yes | Yes | - | - | - | Plasma/Serum | LSCC /NPC | ^9^ |
| Hsa-mir-498 | Yes | Yes | Yes | - | - | - | - | - | - | Not reported in plasma or serum |
| Hsa-mir-181a-5p | Yes | Yes | Yes | Yes | Yes | Yes | - | Plasma  /Serum | OSCC/HNSCC | ^1,8,10^ |
| Hsa-mir-505-5p | Yes | Yes | Yes | - | - | - | - | - | - | Not reported in plasma or serum |
| Hsa-mir-23b-3p | Yes | Yes | Yes | Yes | Yes | Yes | - | - | - | Not reported in plasma or serum |
| Hsa-mir-125b-5p | Yes | Yes | Yes | Yes | - | - | - | Plasma | LSCC | ^9^ |
| Hsa-mir-93-5p | Yes | Yes | Yes | Yes | Yes | Yes | - | Plasma/Serum | HNSCC | ^8,11^ |
| Hsa-mir-371b-5p | Yes | Yes | Yes | - | - | - | - | - | - | Not reported in plasma or serum |
| Hsa-mir-125a-5p | Yes | Yes | Yes | Yes | - | - | - | Plasma | OSCC | ^12^ |
| Hsa-mir-151a-5p | Yes | Yes | Yes | Yes | - | - | Family | Serum | NPC | ^2^ |
| Hsa-mir-320e | Yes | Yes | Yes | - | - | - | - | - | - | Not reported in plasma or serum |
| Hsa-mir-193a-5p | Yes | Yes | Yes | - | - | - | - | - | - | Not reported in plasma or serum |
| Hsa-mir-151a-3p | Yes | Yes | Yes | Yes | - | - | - | Serum | NPC | ^2^ |
| Hsa-mir-31-5p | Yes | Yes | Yes | - | - | - | - | Serum | HNSCC | ^13^ |
| Hsa-mir-107 | Yes | Yes | Yes | - | - | - | - | Serum | OSCC | ^8^ |
| Hsa-mir-425-5p | Yes | Yes | Yes | Yes | - | - | - | Plasma/Serum | HNSCC/NPC | ^3,11^ |
| Hsa-mir-193b-3p | Yes | Yes | Yes | - | - | - | - | Serum | LSCC | ^3^ |
| Hsa-mir-99b-5p | Yes | Yes | Yes | - | Yes | - | - | Plasma/Serum | HNSCC | ^9,13^ |
| Hsa-mir-106b-5p | Yes | Yes | Yes | Yes | Yes | Yes | - | Plasma | HNSCC | ^11^ |
| Hsa-mir-24-3p | Yes | Yes | Yes | Yes | Yes | Yes | - | Plasma/Serum | OSCC/NPC/NPC | ^7,14-16^ |
| Hsa-mir-27b-3p | Yes | Yes | Yes | Yes | - | - | - | Plasma | OSCC/SCC | ^4,17^ |
| Hsa-mir-320d | Yes | Yes | Yes |  |  |  | - | - | - | Not reported in plasma or serum |
| Hsa-mir-22-3p | Yes | Yes | Yes | Yes | - | - | Family | Serum | NPC | ^18^ |
| Hsa-mir-16-5p | Yes | Yes | Yes | Yes | Yes | Yes | - | Plasma/Serum | OSCC/NPC/HNSCC | ^6,7,19^ |
| Hsa-mir-103a-3p | Yes | Yes | Yes | - | - | - | - | Serum | HNSCC | ^8^ |
| Hsa-mir-27a-3p | Yes | Yes | Yes | - | - | - | - | Plasma | OSCC | ^17^ |
| Hsa-mir-210-3p | Yes | Yes | Yes | - | - | - | - | - | - | Not reported in plasma or serum |
| Hsa-mir-574-3p | - | Yes | - | Yes | - | - | - | Plasma | HNSCC | ^20^ |
| Hsa-mir-342-3p | - | Yes | - | Yes | Yes | Yes | - | Serum | NPC | ^3^ |
| Hsa-mir-224-5p | - | Yes | - | - | - | - | - | Serum | HNSCC | ^8^ |
| Hsa-mir-203a | - | Yes | - | - | - | - | - | - | - | Not reported in plasma or serum |
| Hsa-mir-99b-3p | - | Yes | - | - | - | - | Family | Plasma | LSCC | ^9^ |
| Hsa-mir-652-3p | - | Yes | - | Yes | Yes | Yes | - | - | - | Not reported in plasma or serum |
| Hsa-mir-141-3p | - | Yes | - | - | - | - | - | Plasma | NPC | ^6^ |
| Hsa-mir-136-3p | - | Yes | - | - | - | - | Family | Serum | NPC | ^2^ |
| Hsa-mir-486-5p | - | Yes | - | Yes | Yes | Yes | - | Serum | HNSCC | ^3,8^ |
| Hsa-mir-92b-3p | - | Yes | - | - | - | - | - | Plasma/Serum | OSCC/OSCC | ^5,21^ |
| Hsa-mir-3135b | - | Yes | - | - | - | - | - | - | - | Not reported in plasma or serum |
| Hsa-mir-212-3p | - | Yes | - | - | - | - | - | Plasma | LSCC | ^9^ |
| Hsa-mir-23b-5p | - | Yes | - | - | - | - | - | - | - | Not reported in plasma or serum |
| Hsa-mir-151b | - | Yes | - | - | - | - | Family | Serum | NPC | ^2^ |
| Hsa-mir-324-5p | - | Yes | - | Yes | - | - | - | - | - | Not reported in plasma or serum |
| Hsa-mir-18a-5p | - | Yes | - | - | - | - | - | Plasma/Serum | LSCC/OSCC | ^9^ |
| Hsa-mir-378c | - | Yes | - | - | - | - | Family | Plasma | NPC | ^7^ |
| Hsa-mir-187-3p | - | Yes | - | - | - | - | Family | Plasma | OSCC | ^22^ |
| Hsa-mir-30a-3p | - | Yes | - | - | - | - | - | Plasma | OSCC | ^10^ |
| Hsa-mir-194-5p | - | Yes | - | Yes | Yes | Yes | - | Plasma | LSCC | ^9^ |
| Hsa-mir-200a-3p | - | Yes | - | - | - | - | - | - | - | Not reported in plasma or serum |
| Hsa-mir-140-3p | - | Yes | - | Yes | Yes | Yes | - | Serum | NPC | ^3^ |
| Hsa-mir-181d-5p | - | Yes | - | - | - | - | Family | Plasma | OSCC | ^1^ |
| Hsa-mir-132-3p | - | Yes | - | Yes | - | - | - | - | - | Not reported in plasma or serum |
| Hsa-mir-139-5p | - | Yes | - | Yes | Yes | Yes | - | Plasma/Serum | OSCC/SCC/OSCC | ^4,5,10^ |
| Hsa-mir-21-5p | - | Yes | - | - | Yes | - | - | Plasma/Serum | HNSCC/LSCC/NPC/OSCC | ^5,7,9,11,19^ |
| Hsa-mir-155-5p | - | Yes | - | - | - | - | - | Plasma | NPC | ^7^ |
| Hsa-mir-28-5p | - | Yes | - | Yes | - | - | - | Serum | OSCC | ^3,8^ |
| Hsa-mir-138-5p | - | Yes | - | - | - | - | - | Serum | HNSCC | ^13^ |
| Hsa-mir-365a-5p | - | Yes | - | - | - | - | - | - | - | Not reported in plasma or serum |
| Hsa-mir-126-3p | - | Yes | - | Yes | Yes | Yes | - | Serum | NPC | ^23^ |
| Hsa-mir-342-5p | - | Yes | - | - | - | - | - | Plasma | NPC | ^24^ |
| Hsa-mir-877-5p | - | Yes | - | - | - | - | Family | Serum | HNSCC/OSCC | ^5,23^ |
| Hsa-mir-503-5p | - | Yes | - | - | - | - | Family | Serum | HNSCC | ^23^ |
| Hsa-mir-34c-3p | - | Yes | - | - | - | - | - | - | - | Not reported in plasma or serum |
| Hsa-mir-27b-5p | - | Yes | - | - | - | - | - | Plasma | OSCC | ^17^ |
| Hsa-mir-152-3p | - | Yes | - | - | - | - | - | Serum | NPC | ^25^ |
| Hsa-mir-361-5p | Yes | - | - | - | - | - | - | - | - | Not reported in plasma or serum |
| Hsa-mir-30d-5p | Yes | - | - | Yes | - | - | - | Serum | NPC | ^3^ |
| Hsa-mir-665 | Yes | - | - | - | - | - | - | - | - | Not reported in plasma or serum |
| Hsa-let-7b-5p | Yes | - | - | Yes | Yes | Yes | - | Serum | NPC | ^3^ |
| Hsa-let-7f-5p | Yes | - | - | Yes | - | - | - | Serum | HNSCC | ^8^ |
| Hsa-mir-221-3p | Yes | - | - | Yes | Yes | Yes | - | Plasma | LCa | ^26^ |
| Hsa-mir-664a-5p | Yes | - | - | - | - | - | - | - | - | Not reported in plasma or serum |
| Hsa-mir-675-5p | Yes | - | - | - | - | - | - | - | - | Not reported in plasma or serum |
| Hsa-mir-320a | Yes | - | - | Yes | Yes | Yes | - | Serum | HNSCC | ^8^ |
| Hsa-mir-30a-5p | Yes | - | - | Yes | - | - | - | Plasma/Serum | NPC/OSCC | ^3,10^ |
| Hsa-mir-185-5p | Yes | - | - | Yes | - | - | - | - | - | Not reported in plasma or serum |
| Hsa-let-7d-5p | Yes | - | - | Yes | Yes | Yes | Family | Serum | HRLs, CIS or OSCC | ^6^ |
| Hsa-mir-193b-5p | Yes | - | - | - | - | - | - | Serum | NPC | 57 |
| Hsa-let-7i-5p | Yes | - | - | Yes | - | - | - | - | - | Not reported in plasma or serum |
| Hsa-mir-320b | Yes | - | - | Yes | Yes | Yes | - | Serum | OSCC /NPC/HNSCC/NPC | ^6,8,18^ |
| Hsa-let-7a-5p | Yes | - | - | Yes | - | - | - | Plasma/Serum | NPC/HNSCC | ^7,8^ |
| Hsa-mir-211-3p | Yes | - | - | - | - | - | - | - | - | Not reported in plasma or serum |
| Hsa-mir-654-5p | Yes | - | - | - | - | - | - | - | - | Not reported in plasma or serum |
| Hsa-mir-200b-5p | Yes | - | - | - | - | - | Family | Plasma | OSCC | ^2^ |
| Hsa-mir-200c-3p | Yes | - | - | - | - | - | Family | Plasma | SCC | ^4^ |
| Hsa-mir-663a | Yes | - | - | - | - | - | Family | Serum | NPC | ^27^ |
| Hsa-mir-200b-3p | Yes | - | - | - | - | - | - | Plasma | OSCC | ^28^ |
| Hsa-mir-92a-3p | Yes | - | - | Yes | - | - | - | Plasma | HNSCC | ^19^ |
| Hsa-mir-130b-3p | Yes | - | - | Yes | - | - | - | Plasma | OSCC | ^10^ |
| Hsa-let-7c-5p | Yes | - | - |  |  |  | - | Serum | OSCC | ^5^ |
| Hsa-mir-23a-3p | Yes | - | - | Yes | - | - | - | - | - | Not reported in plasma or serum |
| Hsa-mir-485-5p | Yes | - | - | - | - | - | - | - | - | Not reported in plasma or serum |
| Hsa-mir-744-5p | Yes | - | - | - | - | - | Family | Serum | NPC | ^29^ |
| Hsa-mir-196a-5p | Yes | - | - | - | - | - | Family | Plasma/Serum | OSCC | ^5,30^ |
| Hsa-mir-150-3p | Yes | - | - | - | - | - | Family | Plasma | NPC | ^24^ |
| Hsa-mir-25-3p | Yes | - | - | Yes | - | - | - | Plasma | LSCC | ^9^ |
| Hsa-mir-510-5p | Yes | - | - | - | - | - | - | - | - | Not reported in plasma or serum |
| Hsa-mir-564 | Yes | - | - | - | - | - | - | - | - | Not reported in plasma or serum |
| Hsa-mir-25-5p | Yes | - | - | - | - | - | Family | Serum | HRLs, CIS or OSCC | ^6^ |
| Hsa-mir-361 | Yes | - | - | - | - | - | - | - | - | Not reported in plasma or serum |
| Hsa-mir-455-3p | Yes | - | - | - | - | - | - | Plasma | NPC | ^24^ |
| Hsa-mir-423-5p | Yes | - | - | Yes | - | - | - | Plasma/Serum | OSCC/NPC/NPC | ^12,18^ |
| Hsa-mir-920 | Yes | - | - | - | - | - | - | - | - | Not reported in plasma or serum |
| Hsa-mir-135a-3p | Yes | - | - | - | - | - | Family | Serum | NPC | ^31^ |
| Hsa-mir-205-5p | Yes | - | - | - | - | - | - | Plasma | LSCC | ^9^ |
| Hsa-mir-320c | Yes | - | - | - | - | - | - | Plasma | NPC | ^31^ |
| Hsa-mir-29a-3p | Yes | - | - | Yes | - | - | - | Serum | OSCC | ^6^ |
| Hsa-mir-15a-5p | Yes | - | - | - | - | - | - | - | - | Not reported in plasma or serum |
| Hsa-mir-572 | Yes | - | - | - | - | - | - | Serum | NPC | ^31^ |
| Hsa-mir-15b-5p | Yes | - | - | Yes | - | - | - | - | - | Not reported in plasma or serum |
| Hsa-let-7g-5p | Yes | - | - | Yes | Yes | Yes | - | Serum | NPC | ^3^ |
| Hsa-mir-885 | Yes | - | - | - | - | - | Family | Plasma | HNSCC | ^11^ |
| Hsa-mir-664b-5p | Yes | - | - | - | - | - | - | - | - | Not reported in plasma or serum |
| Hsa-let-7d-3p | Yes | - | - | Yes | - | - | - | Serum | NPC | ^3^ |
| Hsa-mir-92b-5p | Yes | - | - | - | - | - | Family | Plasma | OSCC | ^21^ |
| Hsa-mir-602 | Yes | - | - | - | - | - | - | - | - | Not reported in plasma or serum |
| Hsa-mir-20a-5p | Yes | - | - | Yes | - | - | - | Plasma/Serum | NPC/SCC/OSCC | ^4,5,15^ |
| Hsa-mir-106a-5p | Yes | - | - | Yes | - | - | - | Plasma | OSCC | ^10^ |
| Hsa-mir-222-3p | Yes | - | - |  |  |  | - | Plasma | OSCC | ^12^ |
| Hsa-let-7e-5p | Yes | - | - | Yes | - | - | - | Plasma | SCC | ^4^ |
| Hsa-mir-129-5p | Yes | - | - | - | - | - | - | - | - | Not reported in plasma or serum |
| Hsa-mir-943 | Yes | - | - | - | - | - | - | - | - | Not reported in plasma or serum |
| Hsa-mir-100-5p | Yes | - | - | - | - | - | - | Serum | HNSCC | ^8^ |
| Hsa-mir-182-5p | Yes | - | - | - | - | - | - | Serum | NPC | ^2^ |
| Hsa-mir-760 | Yes | - | - | - | - | - | - | - | - | Not reported in plasma or serum |
| Hsa-mir-885-3p | Yes | - | - | - | - | - | - | Plasma | HNSCC | ^11^ |
| Hsa-mir-936 | Yes | - | - | - | - | - | - | - | - | Not reported in plasma or serum |
| Hsa-mir-191-5p | Yes | - | - | Yes | Yes | Yes | - | Plasma/Serum | HNSCC | ^8,20^ |

HNSCC- head and neck squamous cell carcinoma; NPC- nasopharyngeal carcinoma; LSCC-laryngeal squamous cell cancer, OSCC- oral squamous cell carcinoma; HRLs- high risk oral lesions, CIS- oral carcinoma in situ; LCa- larynx cancer; SCC- squamous cell carcinoma; (-) lack of information.

**Supplementary Table S2:** MicroRNAs detected in EVs FaDu and SCC25 and present in plasma of OPHSCC and OSCC pacients in this study and reported in the literature in plasma/serum of HNSCC patients.

**REFERENCES**

1 Yang, C. C. *et al.* miR-181 as a putative biomarker for lymph-node metastasis of oral squamous cell carcinoma. *J Oral Pathol Med* **40**, 397-404, doi:10.1111/j.1600-0714.2010.01003.x (2011).

2 Plieskatt, J. L. *et al.* Methods and matrices: approaches to identifying miRNAs for nasopharyngeal carcinoma. *J Transl Med* **12**, 3, doi:10.1186/1479-5876-12-3 (2014).

3 Zeng, X. *et al.* Circulating miR-17, miR-20a, miR-29c, and miR-223 combined as non-invasive biomarkers in nasopharyngeal carcinoma. *PLoS One* **7**, e46367, doi:10.1371/journal.pone.0046367 (2012).

4 Rabinowits, G. *et al.* Comparative Analysis of MicroRNA Expression among Benign and Malignant Tongue Tissue and Plasma of Patients with Tongue Cancer. *Front Oncol* **7**, doi:10.3389/fonc.2017.00191 (2017).

5 Schneider, A. *et al.* Tissue and serum microRNA profile of oral squamous cell carcinoma patients. *Sci Rep* **8**, 675, doi:10.1038/s41598-017-18945-z (2018).

6 MacLellan, S. A. *et al.* Differential expression of miRNAs in the serum of patients with high-risk oral lesions. *Cancer Med* **1**, 268-274, doi:10.1002/cam4.17 (2012).

7 Liu, X. *et al.* Diagnostic and prognostic value of plasma microRNA deregulation in nasopharyngeal carcinoma. *Cancer Biol Ther* **14**, 1133-1142, doi:10.4161/cbt.26170 (2013).

8 Victoria Martinez, B. *et al.* Circulating small non-coding RNA signature in head and neck squamous cell carcinoma. *Oncotarget* **6**, 19246-19263, doi:10.18632/oncotarget.4266 (2015).

9 Ayaz, L., Gorur, A., Yaroglu, H. Y., Ozcan, C. & Tamer, L. Differential expression of microRNAs in plasma of patients with laryngeal squamous cell carcinoma: potential early-detection markers for laryngeal squamous cell carcinoma. *J Cancer Res Clin Oncol* **139**, 1499-1506, doi:10.1007/s00432-013-1469-2 (2013).

10 Severino, P. *et al.* Small RNAs in metastatic and non-metastatic oral squamous cell carcinoma. *BMC Med Genomics* **8**, 31, doi:10.1186/s12920-015-0102-4 (2015).

11 Summerer, I. *et al.* Changes in circulating microRNAs after radiochemotherapy in head and neck cancer patients. *Radiat Oncol* **8**, 296, doi:10.1186/1748-717x-8-296 (2013).

12 Chang, Y. A. *et al.* A Three-MicroRNA Signature as a Potential Biomarker for the Early Detection of Oral Cancer. *Int J Mol Sci* **19**, doi:10.3390/ijms19030758 (2018).

13 Lu, Z. *et al.* in *Mol Ther Nucleic Acids* Vol. 16 471-480 (2019).

14 Lin, S. C. *et al.* miR-24 up-regulation in oral carcinoma: positive association from clinical and in vitro analysis. *Oral Oncol* **46**, 204-208, doi:10.1016/j.oraloncology.2009.12.005 (2010).

15 Ye, S. B. *et al.* Tumor-derived exosomes promote tumor progression and T-cell dysfunction through the regulation of enriched exosomal microRNAs in human nasopharyngeal carcinoma. *Oncotarget* **5**, 5439-5452, doi:10.18632/oncotarget.2118 (2014).

16 Ye, S. B. *et al.* Exosomal miR-24-3p impedes T-cell function by targeting FGF11 and serves as a potential prognostic biomarker for nasopharyngeal carcinoma. *J Pathol* **240**, 329-340, doi:10.1002/path.4781 (2016).

17 Lo, W. Y., Wang, H. J., Chiu, C. W. & Chen, S. F. miR-27b-regulated TCTP as a novel plasma biomarker for oral cancer: from quantitative proteomics to post-transcriptional study. *J Proteomics* **77**, 154-166, doi:10.1016/j.jprot.2012.07.039 (2012).

18 Liu, N. *et al.* A four-miRNA signature identified from genome-wide serum miRNA profiling predicts survival in patients with nasopharyngeal carcinoma. *Int J Cancer* **134**, 1359-1368, doi:10.1002/ijc.28468 (2014).

19 Poel, D., Buffart, T. E., Oosterling-Jansen, J., Verheul, H. M. & Voortman, J. Evaluation of several methodological challenges in circulating miRNA qPCR studies in patients with head and neck cancer. *Exp Mol Med* **50**, e454, doi:10.1038/emm.2017.288 (2018).

20 Summerer, I. *et al.* Circulating microRNAs as prognostic therapy biomarkers in head and neck cancer patients. *Br J Cancer* **113**, 76-82, doi:10.1038/bjc.2015.111 (2015).

21 Yan, Y. *et al.* Circulating miRNAs as biomarkers for oral squamous cell carcinoma recurrence in operated patients. *Oncotarget* **8**, 8206-8214, doi:10.18632/oncotarget.14143 (2017).

22 Liu, C. J. *et al.* Plasma miR-187* is a potential biomarker for oral carcinoma. *Clin Oral Investig* **21**, 1131-1138, doi:10.1007/s00784-016-1887-z (2017).

23 Liu, C. *et al.* Combined identification of three miRNAs in serum as effective diagnostic biomarkers for HNSCC. *EBioMedicine* **50**, 135-143, doi:10.1016/j.ebiom.2019.11.016 (2019).

24 Wang, S. *et al.* The potent tumor suppressor miR-497 inhibits cancer phenotypes in nasopharyngeal carcinoma by targeting ANLN and HSPA4L. *Oncotarget* **6**, 35893-35907, doi:10.18632/oncotarget.5651 (2015).

25 Zou, X. *et al.* MicroRNA expression profiling analysis in serum for nasopharyngeal carcinoma diagnosis. *Gene* **727**, 144243, doi:10.1016/j.gene.2019.144243 (2020).

26 Yilmaz, S. S. *et al.* MiR-221 as a pre- and postoperative plasma biomarker for larynx cancer patients. *Laryngoscope* **125**, E377-381, doi:10.1002/lary.25332 (2015).

27 Liang, S. *et al.* Increased Serum Level of MicroRNA-663 Is Correlated with Poor Prognosis of Patients with Nasopharyngeal Carcinoma. *Dis Markers* **2016**, 7648215, doi:10.1155/2016/7648215 (2016).

28 Sun, G. *et al.* miR-200b-3p in plasma is a potential diagnostic biomarker in oral squamous cell carcinoma. *Biomarkers*, 1-5, doi:10.1080/1354750x.2017.1289241 (2017).

29 Yu, Q., Zhang, F., Du, Z. & Xiang, Y. Up-regulation of serum miR-744 predicts poor prognosis in patients with nasopharyngeal carcinoma. *Int J Clin Exp Med* **8**, 13296-13302 (2015).

30 Liu, C. J. *et al.* miR-196a overexpression and miR-196a2 gene polymorphism are prognostic predictors of oral carcinomas. *Ann Surg Oncol* **20 Suppl 3**, S406-414, doi:10.1245/s10434-012-2618-6 (2013).

31 Zhang, H. *et al.* Identification of a 7-microRNA signature in plasma as promising biomarker for nasopharyngeal carcinoma detection. *Cancer Med* **9**, 1230-1241, doi:10.1002/cam4.2676 (2020).
