# Supplementary material for: Extracellular vesicles cargo from head and neck cancer cell lines disrupt dendritic cells function and match plasma microRNAs
Source: Sci Rep. 2021 Sep 17;11:18534. doi: 10.1038/s41598-021-97753-y (PMC8448882; doi:10.1038/s41598-021-97753-y)
Supplement: Supplementary file 4 — Supplementary Table S3. [file 41598_2021_97753_MOESM4_ESM.docx]

| **Patient** | **Smoking status** | **Drinking status** | **Gender** | **Age (years)** | **Site** | **Pathologic stage** | | **Sample Type** | **Clinical status of the samples** | **Experiment** |
| --- | --- | --- | --- | --- | --- | --- | --- | --- | --- | --- |
| **BR-001/0039** | Current | Current | Female | 52 | Base of tongue | | T4aN2cM0 | Plasma | Diagnosis | PCR *Array* |
| **BR-001/0057** | Former | Former | Male | 50 | Base of tongue | | T4aN2cM0 | Plasma | Diagnosis | PCR *Array* |
| **BR-001/0074** | Current | Current | Male | 60 | Base of tongue | | T4bN2cM0 | Plasma | Diagnosis | PCR *Array* |
| **BR-001/0109** | Current | Current | Male | 55 | Base of tongue | | T3N0M0 | Plasma | Diagnosis | PCR *Array* |
| **BR-001/0124** | Current | Current | Male | 52 | Base of tongue | | T4aN3M1 | Plasma | Diagnosis | PCR *Array* |
| **BR-001/0223** | Current | Current | Female | 55 | Base of tongue | | T2N0MO | Plasma | Diagnosis | PCR *Array* |
| **BR-001/0250** | Current | Current | Male | 52 | Base of tongue | | T4aN2aM0 | Plasma | Diagnosis | PCR *Array* |
| **BR-001/0256** | Current | Current | Male | 62 | Base of tongue | | T3N0M0 | Plasma | Diagnosis | PCR *Array* |
| **BR-001/0263** | Former | Current | Male | 52 | Base of tongue | | T4aN3M0 | Plasma | Diagnosis | PCR *Array* |
| **BR-001/0285** | Current | Current | Male | 51 | Base of tongue | | T3N3M0 | Plasma | Diagnosis | PCR *Array* |
| **BR-001/0288** | Current | Former | Male | 54 | Base of tongue | | T3N3M0 | Plasma | Diagnosis | PCR *Array* |
| **BR-001/0292** | Former | Former | Male | 66 | Base of tongue | | T4aN2bM0 | Plasma | Diagnosis | PCR *Array* |
| **BR-001/0301** | Former | Former | Male | 51 | Base of tongue | | T4N0M0 | Plasma | Diagnosis | PCR *Array* |
| **BR-001/0304** | Current | Former | Male | 60 | Base of tongue | | T3N2cM0 | Plasma | Diagnosis | PCR *Array* |
| **BR-001/0321** | Current | Former | Male | 54 | Base of tongue | | T2N0M0 | Plasma | Diagnosis | PCR *Array* |
| **BR-001/0328** | Former | Former | Male | 65 | Edge of the tongue | | T2N0M0 | Plasma | Diagnosis | PCR *Array* |
| **BR-001/0046** | Current | Current | Male | 54 | Edge of the tongue | | T4aN2cM0 | Plasma | Diagnosis | PCR *Array* |
| **BR-001/0105** | Current | Former | Female | 80 | Edge of the tongue | | T4aN2cM0 | Plasma | Diagnosis | PCR *Array* |
| **BR-001/0134** | Current | Current | Male | 57 | Edge of the tongue | | T3N2bM0 | Plasma | Diagnosis | PCR *Array* |
| **BR-001/0149** | Current | Current | Male | 58 | Edge of the tongue | | T2N2bM0 | Plasma | Diagnosis | PCR *Array* |
| **BR-001/0151** | Former | Current | Male | 65 | Edge of the tongue | | T2N2cM0 | Plasma | Diagnosis | PCR *Array* |
| **BR-002/0020** | Current | Current | Male | 58 | Edge of the tongue | | T2N0M0 | Plasma | Diagnosis | PCR *Array* |
| **BR-002/0025** | Current | Current | Male | 58 | Edge of the tongue | | T2N1M0 | Plasma | Diagnosis | PCR *Array* |
| **BR-002/0068** | Current | Former | Male | 67 | Edge of the tongue | | T1N0M0 | Plasma | Diagnosis | PCR *Array* |
| **BR-002/0072** | Current | Former | Male | 53 | Edge of the tongue | | T3N0M0 | Plasma | Diagnosis | PCR *Array* |
| **BR-001/0025** | Current | Current | Male | 61 | Mouth floor | | T4aN2cM0 | Plasma | Diagnosis | PCR *Array* |
| **BR-001/0049** | Former | Current | Male | 70 | Mouth floor | | T4aN1cM0 | Plasma | Diagnosis | PCR *Array* |
| **BR-001/0061** | Former | Former | Male | 50 | Mouth floor | | T4aN2cM0 | Plasma | Diagnosis | PCR *Array* |
| **BR-001/0111** | Current | Current | Male | 55 | Mouth floor | | T1N3M1 | Plasma | Diagnosis | PCR *Array* |
| **BR-001/0115** | Current | Former | Male | 56 | Mouth floor | | T4aN2cM0 | Plasma | Diagnosis | PCR *Array* |
| **BR-001/0119** | Current | Current | Male | 50 | Mouth floor | | T4bN3M0 | Plasma | Diagnosis | PCR *Array* |
| **BR-001/0220** | Former | Former | Female | 50 | Mouth floor | | T1N0M0 | Plasma | Diagnosis | PCR *Array* |
| **BR-001/0236** | Current | Current | Male | 53 | Mouth floor | | T4aN2bM0 | Plasma | Diagnosis | PCR *Array* |
| **BR-001/0248** | Former | Former | Male | 55 | Mouth floor | | T2N2aM0 | Plasma | Diagnosis | PCR *Array* |
| **BR-001/0308** | Current | Current | Male | 50 | Mouth floor | | T4aN0M0 | Plasma | Diagnosis | PCR *Array* |
| **BR-001/0323** | Current | Former | Male | 50 | Mouth floor | | T4aN2cM0 | Plasma | Diagnosis | PCR *Array* |
| **BR-001/0335** | Current | Current | Female | 50 | Mouth floor | | T2N0M0 | Plasma | Diagnosis | PCR *Array* |
| **BR-002/0021** | Former | Current | Male | 63 | Mouth floor | | T3N0M0 | Plasma | Diagnosis | PCR *Array* |
| **BR-002/0031** | Current | Former | Male | 62 | Mouth floor | | T1N2bM0 | Plasma | Diagnosis | PCR *Array* |
| **BR-002/0035** | Current | Former | Male | 57 | Mouth floor | | T1N0M0 | Plasma | Diagnosis | PCR *Array* |
| **BR-002/0041** | Current | Former | Male | 64 | Mouth floor | | T4aN0M0 | Plasma | Diagnosis | PCR *Array* |
| **BR-002/0048** | Current | Current | Male | 52 | Mouth floor | | T2N2bM0 | Plasma | Diagnosis | PCR *Array* |
| **BR-002/0058** | Current | Current | Male | 58 | Mouth floor | | T4N2bM0 | Plasma | Diagnosis | PCR *Array* |
| **BR-002/0062** | Current | Current | Male | 55 | Mouth floor | | T1N0M0 | Plasma | Diagnosis | PCR *Array* |
| **BR-002/0095** | Current | Current | Male | 52 | Mouth floor | | T3N2bM0 | Plasma | Diagnosis | PCR *Array* |
|  |  |  |  |  |  | |  |  |  |  |

T- Primary tumor; N- Regional lymph nodes; M- Distant metastasis.

**Supplementary Table S3:** Clinical and pathological data of patients in this study.
